# Diversity of returnee executives’ foreign experience and corporate social responsibility performance

**DOI:** 10.1371/journal.pone.0300262

**Published:** 2024-04-01

**Authors:** Yunyu Wu, Weiguo Zhang, Hua Li

**Affiliations:** 1 Department of Business Management, School of Economics and Business Administration, Chongqing University, Chongqing, People’s Republic of China; 2 College of Economics and Management, Southwest University, Chongqing, People’s Republic of China; Universiti Malaysia Kelantan, MALAYSIA

## Abstract

Top managers’ past experiences (e.g., foreign experience) significantly impact their decision-making behavior, which may influence firms’ sustainable development. The available literature, focusing on the role of the increase in the number of top executives with foreign experience in corporate social responsibility (CSR), yields mixed results. In order to clarify the ambiguous relationship between executive foreign experience and CSR, we empirically examine the effect of the geographic diversity of top executives’ foreign experience on CSR. Based on a hand-collected dataset of the top management team’s (TMT’s) foreign experience, we demonstrate the positive impact of the geographic diversity of returnee executives’ foreign experience on firms’ CSR using Chinese A-share listed firms from 2009 to 2018. Moreover, this impact is stronger in firms with political connections with the central government and in regions with good market development. Furthermore, the mechanism analysis shows that returnee executives drive firms’ CSR by promoting corporate donations and green innovation. This paper offers clear policy implications by suggesting that hiring returnees with a broad geographic scope of foreign experience as corporate executives is an efficient way to enhance firms’ CSR.

## Introduction

As a growing number of individuals return to their countries of origin after gaining education and/or work experience abroad, countries such as China and India are increasingly being depicted as experiencing a “brain gain” rather than a “brain drain” [1]. This form of cross-border mobility has stirred the curiosity of numerous international business scholars, leading to a boom in research on the role that returnees play in transforming the business environment in their home countries. Thus, a substantial body of literature has explored the impact of returnees on new venture creation [2–4] and technological innovation [1, 5]. Nevertheless, limited attention has been paid to the role of returnees in disseminating business practices focused on the social development (e.g., CSR) of their home countries.

Corporate social responsibility (CSR) is generally defined as context-specific organizational actions and policies that account for stakeholder expectations and the triple bottom line of economic, social, and environmental performance [6]. In recent decades, academic research has increasingly focused on CSR and its determinants [e.g., 7–11]. Some scholars have recently directed their attention to the foreign experience of returnee executives with the emergence of the “brain gain” phenomenon. They observed that returnee executives’ foreign experience had a significant impact on CSR’s overall performance [12–16] and its components [17–21].

However, the existing literature regarding returnee executives and CSR produces mixed results. Research has shown that returnee executives benefit firms’ CSR initiatives [12], while it has been suggested that these executives’ foreign experiences hinder the company’s efforts in social action [20]. One explanation for the conflicting results may be that current research focuses solely on the number of returnees and fails to consider the impact of their foreign experience’ characteristics, such as geographic diversity. Geographic diversity represents the geographical scope of a returnee executive’s foreign experience and reflects the breadth and variety of their international knowledge and experience. Previous studies indicate that the variety of foreign exposure locations influences returnee executives’ decision-making behavior, thus resulting in distinct organizational consequences [22–24]. Consequently, geographic diversity may play a key role in elucidating the equivocal association between returnee executives and corporate social performance while comprehensively gauging their worth to firms.

To clarify this uncertain relationship, we investigated the effect of the diversity of returnee executives’ location on CSR using a panel of Chinese-listed companies from 2009 to 2018. Our findings reveal a positive relationship between geographic diversity and firms’ CSR based on ordinary least squares (OLS) regressions. This relationship is more substantial when the regions where firms operate have good market development and when firms have political connections with the central government. Furthermore, we employed a heteroskedasticity-based identification approach [25] to alleviate endogenous concerns. We conducted multiple robustness checks to validate our results. In addition, mechanism analysis indicates that having returnee executives from diverse geographic regions enhances firms’ CSR performance by facilitating corporate donations and promoting green innovations.

Our study makes several contributions to the current literature. First, this study adds to the literature on returnees and corporate social behavior. We investigated the role of foreign experience’ breadth, which is a departure from previous studies that merely focused on the number of returnees. Our findings help clarify the ambiguous relationship between returnees and corporate social action. Second, this paper enhances comprehension of international knowledge transfer’s organizational and institutional boundary conditions. In addition, this paper sheds light on the channel of returnee executives promoting corporate social development. Unlike previous studies concentrating on the direct impact of returnees on corporate sustainable development, this study reveals that returnee talents indirectly impact corporate social performance by promoting corporate donations and green innovations.

## Hypotheses development

### The geographic diversity of returnee executives’ foreign experience and CSR

Geographic diversity represents the geographical scope of returnee executives’ foreign experience, typically operationalized as the number of countries in which returnee executives have studied or worked abroad [22, 26]. The level of geographic diversity reflects, to some extent, the breadth and variety of returnee executives’ international experience and knowledge. We argue that there is a positive relationship between the geographic diversity of returnee executives’ foreign experience and firms’ CSR performance.

At the individual level, returnee executives with a greater geographic diversity of foreign experience will develop more international knowledge and general competencies [22]. Countries vary in their economic, political, social and cultural systems, practices and behaviors. Studying and working in multiple countries exposes returnee executives to various manifestations of foreign environments, which furnishes them with knowledge and competencies transferrable to different contexts. Moreover, international knowledge and competencies, especially those concerning CSR, gained from more countries are of greater value than those gained from fewer countries. In particular, knowledge and competencies derived from multiple countries generally involve a greater degree of causal ambiguity and social complexity [27]. In this vein, the CSR knowledge gained from multiple countries is more global and can be used across different countries rather than a single country [28]. In addition, a broad scope of foreign experience enhances returnee executives’ general cognitive competencies, such as tolerance of difference, multiple worldviews, open-mindedness, and empathy [29–31]. With the enhanced knowledge and competencies described above, returnee executives with broad foreign experience can consider a wide range of solutions when approaching a CSR decision problem [22, 32] and recombine elements of their knowledge to create new insights and solutions [33–35].

At the TMT level, TMTs with a high geographic diversity of foreign experience can notice and interpret a broader range of environmental stimuli [26, 32, 34] and can make a higher-quality CSR decision, resulting in better CSR performance. As we mentioned above, TMTs with a broad scope of foreign experience are expected to have greater international knowledge and general competencies [22], thus, as well as have an enhanced ability to process complex and dynamic information [36]. They can also better understand the various CSR realities in the specific context of China, given their extensive knowledge base and diversified experience concerning CSR practices. As a result, TMTs with extensive foreign experience could potentially consider more alternatives when deciding on CSR, which is more likely to result in more comprehensive, higher-quality CSR decisions and, thus, better CSR performance. By comparison, TMTs with narrow foreign experiences are likely to have a restricted perspective and limited knowledge base from which to search for alternatives [37], especially when faced with an unprecedented problem concerning CSR.

In addition, high levels of geographic diversity can alleviate the risk of misapplying international knowledge to CSR decisions by the TMT with returnee executives. Because experiences from different countries are heterogeneous, there may be a risk that returnee executives erroneously transfer experiential knowledge gained in a host country to an experience or decision concerning the home country [38–40]. This risk is enhanced when returnee executives have a limited geographic scope of foreign experience. Furthermore, the limited scope of experience may hamper the TMT’s ability to apply prior experiential knowledge when deciding on a CSR investment while increasing the risk of overconfidence [41]. Literature shows that managerial overconfidence negatively affects CSR activities [42]. However, the broad experience can enhance TMTs’ awareness of cross-country differences [43] and help them discern what experiential knowledge they can and cannot draw on for a particular CSR decision. This helps avoid incorrect analogizing and erroneous transfer of prior learning [44, 45]. Based on the above analysis, we propose:

Hypothesis 1 (H1). The geographic diversity of returnee executives’ foreign experience is positively related to firms’ CSR.

### Moderators: Corporate political connections and regional market development

The ability of companies to integrate knowledge from multiple countries varies [46]. In this vein, the effect of the geographic diversity of returnee executives’ foreign experience on CSR may be moderated by the organizational characteristic (e.g., corporate political connections) and institutional environment (e.g., regional market development) of the region where the firm is located.

#### The moderating role of corporate political connections

Political connections are commonplace in China and significantly influence firm behavior [47]. Political connections are broadly defined as connections between firms and government agencies or government officials [48]. Typically, Chinese firms build political connections by appointing former or current government bureaucrats as top executives [49]. The prior work experience of these executives serves as a channel of communication and access to existing government officials [50] and endows firms with inside information about government operations and bureaucracy [51]. Prior studies identify that political connections are an important determinant of firms’ CSR involvement [52, 53]. In this paper, we contend that political connections magnify the effect of the geographic diversity of returnee executives’ foreign experience on CSR.

Specifically, political connections compensate for the disadvantage of TMT with returnee executives by providing unique information advantages to the firm, thereby increasing the effectiveness of firms’ CSR investments and improving CSR performance. Although much of the literature emphasizes the value of the human and social capital that returnee executives have accumulated abroad, they also have some drawbacks. In particular, returnee executives have usually lived abroad for a long time, and after returning, they may face a different environment [1] and lack local knowledge and domestic social networks [1, 54]. Therefore, they may not have an accurate understanding of the domestic market environment or access to information about social and political expectations [1, 54], resulting in a lack of sensitivity and knowledge to identify CSR priorities among multiple options. These disadvantages may make them less effective in providing practical advice and formulating a strategic CSR plan and action.

By contrast, politically connected executives are more likely to have local information and connections due to their prior work experience in government agencies. They can endow firms with inside information about government operations and bureaucracy [51] because their prior work experience serves as a key channel for communication and access to existing government agents [50]. In this sense, executives with political connections are better equipped to identify the salient concerns of key stakeholders in the Chinese context, such as the pressing issues facing politicians. In doing so, they aptly compensate for the aforementioned disadvantages of returnee executives by providing “political intelligence” to the firm, thereby increasing the TMT’s sensitivity to salient social issues [55]. Therefore, politically connected executives help TMTs with returnee executives to identify CSR priorities among multiple options, thereby strengthening the effectiveness of their CSR investments. In sum, political connections promote the positive impact of returnee executives on CSR by creating an information advantage for the firm.

Hypothesis 2 (H2). Corporate political connections will strengthen the positive effect of the geographic diversity of returnee executives’ foreign experience on CSR.

#### The moderating role of regional market development

While China has made major progress in marketization, the degree of that progress varies across regions [56]. In some provinces, such as Zhejiang and Jiangsu, markets are more developed with limited government intervention [57]. As a result, firms have more autonomy and freedom in their operations. However, in regions with less developed market structures, the government still exerts considerable control over the economy [58]. Under such circumstances, firm behavior is heavily influenced by the government, a firm’s managerial discretion is weakened, and the influence of top executives on firm outcomes is also constrained [59, 60]. We contend that the increased level of regional market development grants companies more managerial autonomy, thus amplifying the influence of returnee executives on CSR.

In more marketized regions, returnee executives have more managerial discretion and, thus, greater influence on firms’ CSR. Generally, regions with a high level of marketization have a higher quality of market development and better legal infrastructure, such as rigorous contract enforcement and good property rights protection, rendering the distribution of social resources more equitable [49]. Good market development reduces government intervention in economic activities, contributes to market liberalization, and consequently increases the tendency of firms to conform to market rules in making management decisions. At the same time, the reduction of government intervention and the improvement of the market environment also decrease the external constraints on firms and increase their latitude of action [61]. Thus, in more market-oriented regions, TMTs with returnee executives have more managerial discretion and are more likely to spend resources on CSR practices based on their preferences and values [62].

In contrast, in less market-oriented regions with more government intervention and administrative harassment, returnee executives have relatively low managerial discretion and thus less influence on firms’ CSR [49]. Low levels of market development expand the power of the government in economic activities and increase a firm’s external constraints, thereby reducing firms’ latitude of action. The upper-echelon literature shows that TMTs’ characteristics are less likely to be expressed in the form of their actual actions when they lack managerial discretion [20, 63, 64]. Hence, in regions with low levels of market development, TMTs with returnee executives have a reduced role in management decisions, and their influence on corporate social responses is diminished. Therefore, we posit that the level of regional market development exerts a positive moderating influence on the relationship between the geographic diversity of returnee executives’ foreign experience and firms’ CSR.

Hypothesis 3 (H3). As the regional marketization level increases, the positive effect of the geographic diversity of returnee executives’ foreign experience on firms’ CSR will be enhanced.

## Methodology

### Data and sample

We selected Chinese-listed firms that disclosed CSR reports on the Shanghai or Shenzhen Stock Exchange from 2009–2018 and were included in Rankins CSR Rating (RKS) as the original sample. The sample starts from 2009 because the Shanghai and Shenzhen Stock Exchanges—both under the control of the central government—started requiring firms to report on their CSR performance in their annual reports in 2008. The sample ends in 2018 because, after that year, the outbreak of the COVID-19 epidemic led to a surge in CSR investment by Chinese companies. Following normal practice, we exclude (1) financially distressed firms, such as ST (special treatment) firms or negative-equity firms; (2) firms in the financial industry due to such firms’ unique assets structure and accounting system; (3) firms with missing information on key variables. All continuous variables were winsorized at the 1st and 99th percentiles to control for the effect of outliers. We ended up with an unbalanced sample of 776 unique firms for 4,844 firm-year observations.

CSR data were provided by RKS (http://www.rksratings.cn/), a third-party CSR rating agency in China and one of the main sources for ranking Chinese companies’ CSR practices. Firm-level information was obtained from the China Stock Market and Accounting Research (CSMAR) database (https://www.gtarsc.com/). The CSMAR database is the main source for research on Chinese-listed firms, providing reliable information about companies’ backgrounds and financial statistics, and has been widely used in management research. We manually collected data regarding senior executives’ biographies based on their resumes disclosed in firms’ annual reports. We double-checked that information against Sina Finance (https://finance.sina.com.cn/). Data concerning regional market development was obtained from the Marketization Index of China’s Provinces: NERI Report 2021, co-authored by Wang et al. [58].

### Measurements

#### Dependent variable

We utilized the social responsibility scores provided by the RKS to measure a firm’s CSR engagement (*CSR_Score*), similar to studies that use the KLD (Kinder, Lydenberg, Domini & Co., Inc.) score as an indicator of CSR engagement for US firms [65, 66]. RKS adopts a structured expert scoring method, with a full score of 100. The higher the social responsibility score in the RKS, the better the firm’s CSR performance. RKS data have been used extensively in CSR studies in China [52, 67–69] to measure firms’ CSR performance. Validity tests of this measure, conducted by studies [70, 71], also suggest that the RKS data represent the substantive CSR performance of firms.

#### Explanatory variable

Returnee executives are defined as natives of mainland China who have studied or worked in developed regions, including developed countries and Hong Kong, Macau, and Taiwan provinces in China (See S1 Table for a detailed list), and then return to their home country as senior executives in domestic firms. With reference to prior studies [22, 26], we measure the geographic diversity of executive foreign experience as the average number of developed countries where returnee executives studied or worked abroad (*Geographic diversity*).

#### Moderating variables

We measured the regional market development (*MarketDev*) using the Marketization Index of China’s Provinces: NERI Report 2021 [58]. Higher levels of marketization are associated with better regional market development and lower levels of government involvement.

Following previous studies [52, 72], we defined a company as politically connected if at least one of its directors or top managers has served as a government official in central or local government agencies. Thus, to measure firms’ political connections, we first examined the CVs of all TMT and board members to determine whether they have worked in central or local government agencies. Given the differences in motivations, goals, and priorities [73, 74], we created two variables (i.e., *CentralPC* and *LocalPC*) to measure firms’ political connections to the central and local governments, respectively [75, 76]. Central political connection (*CentralPC*) was measured as the number of TMT and board members who have held a national-level principal or deputy position. Local political connection (*LocalPC*) was measured as the number of TMT and board members who have served as local government officials at the division (chu) level or above. Notably, the hierarchy of local officials in China consists of ministry (bu), department (ju), division (chu), section (ke), staff member (keyuan), and clerk (banshiyuan) in descending order. Lower-level government officials, i.e., those below the level of division (chu), are not included in this study because they are not funded through the central financial system and thus tend not to be counted as political elites in China [77].


$$Growth_{it}=MarketIndex_{it}/MarketIndex_{it-1}-1$$

#### Control variables

Consistent with previous research, we included the conventional firm-level variables: return on assets, equity concentration, leverage, firm ownership, firm age, and firm size. To control for board-level governance effects, we also included a set of corporate governance variables: the board size, the percentage of independent directors on the board, whether the chairman and CEO are in one, and whether there are returnee directors. We also considered whether a company has female executives to control for the extent to which executive gender affects a company’s CSR-related practices. We added the average age of the executive team members to control for the positive effect of executive age on CSR-related activities. Additionally, to control for the effect of foreign nationality, we accounted for whether a firm has a foreigner as an executive. Finally, we included *Industry* dummies to control for industry-specific effects and *Year* dummies to control for the omitted variables that vary over time but are constant across firms. All definitions of the dependent, explanatory, moderating, and control variables used in the analyses are presented in Table 1.

**Table 1:** Definitions of variables.

Variable	Definition
CSR_Score	CSR_Score is measured by the social responsibility scores provided by RKS. The detailed evaluation system is shown in S2 Table.
Geographic diversity	The average number of developed countries where returnee executives studied or worked abroad.
MarketDev	MarketDev is measured by Marketization Index of China’s Provinces: NERI Report 2018.
CentralPC	The number of TMT and board members who have worked in central-level government agencies.
LocalPC	The number of TMT and board members who have served as local government officials at division (chu) level or above.
ROA	Net profit/average net assets. Where: average net assets = (number of owners’ equity at the beginning of the year + number of owners’ equity at the end of the year)/2.
Leverage	Liabilities/assets.
Equity concentration	The sum of the shareholding ratios of the top ten shareholders.
Firm size	The natural logarithm of total assets.
Firm age	The number of years of listing.
SOE	1 = state-owned enterprises, 0 = non-state-owned enterprises
Board size	The total number of directors on the board.
Board independence	The proportion of independent directors is computed as the number of independent directors divided by the total number of directors on the board.
Duality	A binary variable equals 1 if the chairman and CEO are in one, otherwise 0.
Female executive	A dummy variable is equal to 1 if there is a female in the TMT and 0 otherwise.
Executive age	The average age of executive team members is calculated as the sum of the ages of all TMT members divided by the total number of TMT members.
Foreigner	A binary variable takes the value of 1 if there is an executive with non-China (mainland) in the TMT and 0 otherwise.
Returnee director	A dummy variable is equal to 1 if there is a director who has studied or worked outside (mainland) China in the board of directors and 0 otherwise.

### Estimation model

We tested our hypotheses with OLS multiple regressions using a dataset consisting of up to ten years of data for each firm. The estimation models are as follows:

$$CSR\_Score_{i,t+1}\;=\beta_{0}+\beta_{1}Geographic\;diversity_{i,t}+Controls_{i,t}+\varepsilon_{i,t}$$ (1)

$$CSR\_Score_{i,t+1}=\beta_{0}+\beta_{1}Geographic\;diversity_{i,t}+\beta_{2}Geographic\;diversity_{i,t}\times Political\;connections+\beta_{3}Geographic\;diversity_{i,t}\times MarketDev_{i,t}+Controls_{i,t}+\varepsilon_{i,t}$$ (2)

The subscript i denotes the firm; t denotes the year. Eq (1) is used to test Hypothesis 1, and Eq (2) is used to test Hypotheses 2 and 3. Where *CSR_Score* is the dependent variable, *Geographic diversity* is the independent variable, and *Political connections* represent the corporate political connections with the central government (*CentralPC*) or local government (*LocalPC*). *MarketDev* represents the level of market development in the region where the company is located. *Controls* is a set of the firm- and executive-level control variables, and *ε* is an error term clustered at the firm level. The industry and year dummies are included to control for fixed industry-specific, time-varying characteristics. Accounting for the potential endogeneity of reverse causality, we lag one year between the dependent variable (t + 1) and the independent variables (t) in all estimations [78].

## Results

### Main results

Table 2 shows the firm-level data set used for the empirical analysis. The average value of CSR_Score is 38.797, with a total score of 100 points, which does not reach 60, indicating that the overall quality of Chinese firms’ CSR is poor. The standard deviation of CSR performance is as high as 12.07, showing that the level of social responsibility among firms is uneven and individual differences are enormous. The correlation matrix is reported in Table 3. We find that the geographic diversity of returnee executives’ foreign experience is positively correlated with firms’ CSR. All the correlations among the control variables are relatively low. Additionally, we calculate the variance inflation factor (VIF) for the control variables, and the highest VIF is 1.95, which is well below the critical value of 10. Therefore, multicollinearity is not a serious concern [79].

**Table 2:** Descriptive statistics.

Variables	Mean	Median	SD	N
CSR_Score	38.797	36.14	12.07	4844
Geographic diversity	0.220	0	0.543	4844
MarketDev	7.784	7.94	1.721	4844
CentralPC	0.275	0	0.62	4844
LocalPC	2.425	2	2.436	4844
ROA	0.09	0.09	0.099	4844
Leverage	0.497	0.51	0.196	4844
Equity concentration	58.89	59.07	16.288	4844
Firm age	12.714	13	5.865	4844
Firm size	23.043	22.9	1.42	4844
Board size	10.755	10	2.769	4844
Board independence	0.381	0.364	0.073	4844
Duality	0.176	0	0.381	4844
Female executive	0.585	1	0.493	4844
Executive age	47.554	47.714	3.533	4844
Foreigner	0.065	0	0.247	4844
Returnee director	0.588	1	0.492	4844
SOE	0.648	1	0.478	4844

Note. The main characteristics of the executive-level data are reported in S1 Table. Industry distribution of sample firms is reported in S3 Table.

**Table 3:** Correlation matrix.

**Variables**	**(1)**	**(2)**	**(3)**	**(4)**	**(5)**	**(6)**	**(7)**	**(8)**	**(9)**
(1)CSR_Score									
(2)Geographic diversity	0.203***								
(3)CentralPC	0.105***	0.129***							
(4)LocalPC	0.084***	0.041***	0.331***						
(5)MarketDev	0.192***	0.109***	-0.032**	-0.091***					
(6)ROA	0.039***	0.012	0.125***	0.079***	0.068***				
(7)Leverage	0.121***	0.010	0.056***	0.073***	-0.016	-0.139***			
(8)Equity concentration	0.275***	0.061***	0.126***	0.152***	0.092***	0.138***	0.018		
(9)Firm age	0.070***	-0.032**	-0.099***	-0.147***	0.084***	-0.090***	0.200***	-0.288***	
(10)Firm size	0.485***	0.174***	0.246***	0.246***	0.122***	0.084***	0.503***	0.326***	0.191***
(11)Board size	0.182***	0.052***	0.128***	0.241***	-0.045***	-0.054***	0.111***	0.089***	0.075***
(12)Board independence	0.040***	0.025*	0.069***	0.106***	0.039***	0.032**	0.002	0.036**	-0.066***
(13)Duality	-0.007	0.049***	0.021	-0.072***	0.128***	0.038***	-0.095***	-0.030**	-0.111***
(14)Female executive	0.060***	0.065***	-0.025*	-0.033**	0.105***	0.042***	-0.094***	-0.081***	0.046***
(15)Executive age	0.275***	0.043***	0.027*	0.101***	0.137***	-0.079***	0.131***	0.158***	0.209***
(16)Foreigner	0.080***	0.105***	-0.008	-0.065***	0.080***	0.037***	-0.060***	0.038***	-0.105***
(17)Returnee director	0.189***	0.249***	0.074***	0.054***	0.191***	0.082***	0.011	0.145***	-0.002
(18)SOE	0.131***	-0.055***	-0.007	0.173***	-0.132***	-0.107***	0.226***	0.114***	0.256***
**Variables**	**(10)**	**(11)**	**(12)**	**(13)**	**(14)**	**(15)**	**(16)**	**(17)**	
(11)Board size	0.234***								
(12)Board independence	0.070***	-0.074***							
(13)Duality	-0.071***	-0.071***	0.074***						
(14)Female executive	-0.071***	-0.031**	0.049***	0.101***					
(15)Executive age	0.353***	0.115***	0.030**	-0.059***	-0.131***				
(16)Foreigner	-0.029**	-0.007	0.036**	0.168***	0.024*	0.009			
(17)Returnee director	0.199***	0.166***	0.049***	0.023	0.050***	0.061***	0.109***		
(18)SOE	0.300***	0.208***	-0.105***	-0.248***	-0.138***	0.333***	-0.178***	-0.043***	

Note. N = 4844.

***, **, * represents p < 0.01, p < 0.05, and p < 0.1, respectively.

Table 4 shows the regression results for testing Hypotheses 1–3. Model 1 includes only the independent variable. Model 2 includes only the control variables. Model 3 includes the geographic diversity of returnee executives’ foreign experience and all the control variables. We find that the coefficient of *Geographic diversity* is significantly positive. The results corroborate H1, postulating a positive relationship between the geographic diversity of returnee executives’ foreign experience and firms’ CSR.

**Table 4:** The effect of the geographic diversity of returnee executives’ foreign experience on firms’ CSR.

Model No.	Model 1	Model 2	Model 3	Model 4	Model 5	Model 6	Model 7
Geographic diversity (H1)	4.007***		1.846***	1.506***	1.809***	1.447**	1.028
	(5.448)		(3.115)	(2.616)	(3.112)	(2.267)	(1.632)
Geographic diversity*CentralPC (H2)				1.522**			1.678**
				(2.412)			(2.212)
CentralPC				0.036			0.098
				(0.068)			(0.186)
Geographic diversity*LocalPC (H2)					0.189		-0.020
					(1.013)		(-0.101)
LocalPC					-0.051		-0.064
					(-0.379)		(-0.482)
Geographic diversity*MarketDev (H3)						0.676*	0.763**
						(1.780)	(1.995)
MarketDev						0.275	0.273
						(1.186)	(1.186)
ROA		2.005	2.371	2.298	2.308	2.191	2.140
		(0.807)	(0.963)	(0.937)	(0.939)	(0.887)	(0.871)
Leverage		-4.950**	-4.749**	-4.538**	-4.763**	-4.795**	-4.645**
		(-2.457)	(-2.396)	(-2.305)	(-2.405)	(-2.434)	(-2.376)
Equity concentration		0.075***	0.077***	0.077***	0.077***	0.076***	0.075***
		(3.133)	(3.240)	(3.229)	(3.221)	(3.181)	(3.161)
Firm age		-0.083	-0.075	-0.067	-0.075	-0.072	-0.066
		(-1.185)	(-1.089)	(-0.977)	(-1.074)	(-1.041)	(-0.950)
Firm size		3.764***	3.603***	3.545***	3.611***	3.602***	3.560***
		(10.200)	(10.024)	(9.690)	(9.750)	(10.106)	(9.614)
Board size		0.166	0.168	0.161	0.179*	0.181*	0.182*
		(1.595)	(1.619)	(1.523)	(1.690)	(1.777)	(1.728)
Board independence		-2.840	-2.661	-2.662	-2.527	-2.480	-2.295
		(-0.912)	(-0.865)	(-0.884)	(-0.822)	(-0.815)	(-0.767)
Duality		-0.196	-0.240	-0.228	-0.226	-0.311	-0.308
		(-0.306)	(-0.381)	(-0.363)	(-0.360)	(-0.494)	(-0.493)
Female executive		2.224***	2.089***	2.108***	2.112***	2.087***	2.115***
		(3.558)	(3.355)	(3.404)	(3.401)	(3.383)	(3.459)
Executive age		0.143	0.145	0.140	0.148	0.146	0.145
		(1.496)	(1.511)	(1.467)	(1.551)	(1.536)	(1.548)
Foreigner		3.209***	2.956**	2.977**	3.005**	2.823**	2.810**
		(2.667)	(2.501)	(2.537)	(2.534)	(2.368)	(2.364)
Returnee director		1.068*	0.676	0.770	0.686	0.648	0.761
		(1.841)	(1.158)	(1.329)	(1.178)	(1.097)	(1.299)
SOE		0.906	1.045	0.997	1.050	1.073	1.031
		(1.164)	(1.364)	(1.308)	(1.366)	(1.397)	(1.348)
Constant	37.914***	-59.251***	-56.188***	-54.400***	-56.278***	-56.030***	-55.364***
	(96.577)	(-7.388)	(-7.141)	(-6.802)	(-6.903)	(-7.180)	(-6.816)
Industry FE	yes	yes	yes	yes	yes	yes	yes
Year FE	yes	yes	yes	yes	yes	yes	yes
Observations	4844	4844	4844	4844	4844	4844	4844
Adjusted R-squared	0.182	0.364	0.370	0.373	0.370	0.373	0.376
F	29.69***	19.18***	18.93***	17.13***	16.74***	17.34***	14.80***

Note. Robust t-statistics (in parentheses) are based on the standard errors clustered by firm to address potential serial correlations in the residuals.

*** p<0.01,

** p<0.05,

* p<0.1.

In addition, we constructed three interaction terms (i.e., Geographic diversity*CentralPC, Geographic diversity*LocalPC, and Geographic diversity*MarketDev) to test H2 and H3. The interaction term in Model 4 is significantly positive, suggesting that corporate political connections with the central government strengthen the effect of returnee executives on CSR. The interaction term in Model 5 is insignificant, indicating that corporate political connections with the local government do not significantly affect the relationship between the geographic diversity of returnee executives’ foreign experience and CSR. Thus, H2 is partially supported. These results echo some previous studies, which find that different levels of political connections play prominent but different roles in explaining firms’ CSR-related practices [52, 72].

The interaction term in Model 6 is only significantly positive at the 10% level, which produces weak evidence for supporting H3. Thus, the positive effect of returnee executives on firms’ CSR may be marginally strengthened as the level of regional marketization increases. Consistent with the view that institutional development reduces firms’ external constraints and thus enhances their latitude of action [61, 80], this finding suggests that good institutional environment helps returnee executives to improve firms’ CSR.

### Robustness checks

Endogeneity problem. Given the inability to find appropriate external instruments, we resort to a novel approach based on heteroskedasticity identification to alleviate the problem of reverse causality or omitted variable bias [25]. This approach does not require external instrumental variables but instead generates instruments from the existing model exploiting the heterogeneity in the error of the first-stage regression. The results in Table 5 support the hypothesis that the geographic diversity of returnee executives’ foreign experience positively impacts firms’ CSR. In a non-tabulated analysis, we conducted White and Breusch-Pagan tests and affirmed the existence of heteroskedasticity. The Kleibergen-Paap rk LM statistic and the Hansen J test verify that the estimated results are not subject to under- and over-identification bias. The Cragg-Donald Wald F statistic is much larger than the Stock–Yogo critical value (at 10% maximum IV size), indicating that our estimation does not suffer from weak instruments. Overall, we present evidence that the main results are robust.

**Table 5:** Endogenous test results.

Model No.	Model 1
Geographic diversity	2.802***
	(3.852)
ROA	-3.270
	(-1.363)
Leverage	-6.989***
	(-3.768)
Equity concentration	0.088***
	(3.708)
Firm age	0.049
	(0.780)
Firm size	3.668***
	(10.394)
Board size	0.268**
	(2.411)
Board independence	-0.674
	(-0.212)
Duality	0.054
	(0.078)
Female executive	2.296***
	(3.646)
Executive age	0.387***
	(4.123)
Foreigner	3.054**
	(2.546)
Returnee director	0.703
	(1.153)
SOE	-0.334
	(-0.443)
Constant	-71.168***
	(-10.113)
Observations	4844
Centered R-squared	0.307
F	30.58***
Under-identification test (Kleibergen-Paap rk LM statistic)	67.292***
Cragg-Donald Wald F statistic	378.417***
Stock-Yogo critical value (at 10% maximal IV size)	11.52
Hansen J	0.321

Note. Robust z-statistics (in parentheses) are based on the standard errors clustered by firms to address potential serial correlations in the residuals.

*** p<0.01,

** p<0.05,

* p<0.1.

In addition, we re-examined the main hypotheses using several robustness checks. First, we used the number of developed countries involved in TMT’s foreign experience (*Geographic diversity1*) as an alternative measure of the geographic diversity of returnee executives’ foreign experience. The results in Table 6 are similar to the previous analysis (See Table 4), indicating that TMTs with a larger scope of foreign experience significantly improve firms’ CSR than those whose members have narrower foreign experiences.

**Table 6:** Robustness checks for an alternative measure of the geographic diversity of returnee executives’ foreign experience.

Model No.	Model 1	Model 2	Model 3	Model 4	Model 5
Geographic diversity1	1.243***	1.017**	1.226***	0.889*	0.551
	(2.768)	(2.400)	(2.828)	(1.827)	(1.150)
Geographic diversity1*CentralPC		0.735**			0.977**
		(2.090)			(2.513)
CentralPC		0.059			0.113
		(0.110)			(0.209)
Geographic diversity1*LocalPC			0.058		-0.076
			(0.470)		-0.076
LocalPC			-0.048		-0.058
			(-0.359)		(-0.432)
Geographic diversity1*MarketDev				0.506*	0.602**
				(1.922)	(2.280)
MarketDev				0.278	0.289
				(1.201)	(1.249)
ROA	2.443	2.442	2.457	2.341	2.373
	(0.994)	(0.993)	(1.000)	(0.951)	(0.965)
Leverage	-4.786**	-4.636**	-4.810**	-4.811**	-4.731**
	(-2.407)	(-2.342)	(-2.415)	(-2.431)	(-2.401)
Equity concentration	0.076***	0.076***	0.076***	0.075***	0.076***
	(3.188)	(3.202)	(3.175)	(3.149)	(3.164)
Firm age	-0.077	-0.070	-0.078	-0.074	-0.069
	(-1.115)	(-1.012)	(-1.115)	(-1.073)	(-0.989)
Firm size	3.586***	3.540***	3.597***	3.588***	3.561***
	(9.959)	(9.668)	(9.660)	(10.038)	(9.571)
Board size	0.168	0.161	0.178*	0.182*	0.180*
	(1.621)	(1.532)	(1.684)	(1.781)	(1.714)
Board independence	-2.733	-2.832	-2.620	-2.589	-2.489
	(-0.890)	(-0.945)	(-0.855)	(-0.851)	(-0.836)
Duality	-0.265	-0.281	-0.259	-0.319	-0.361
	(-0.421)	(-0.446)	(-0.412)	(-0.507)	(-0.575)
Female executive	2.127***	2.172***	2.149***	2.117***	2.164***
	(3.415)	(3.511)	(3.461)	(3.429)	(3.540)
Executive age	0.149	0.142	0.151	0.151	0.147
	(1.554)	(1.487)	(1.590)	(1.588)	(1.564)
Foreigner	2.954**	2.994**	2.973**	2.846**	2.822**
	(2.492)	(2.534)	(2.500)	(2.388)	(2.362)
Returnee director	0.725	0.802	0.729	0.695	0.802
	(1.239)	(1.385)	(1.250)	(1.176)	(1.371)
SOE	1.027	1.022	1.036	1.047	1.045
	(1.338)	(1.336)	(1.346)	(1.360)	(1.362)
Constant	-55.842***	-54.279***	-56.009***	-55.872***	-55.368***
	(-7.090)	(-6.788)	(-6.834)	(-7.129)	(-6.786)
Industry FE	yes	yes	yes	yes	yes
Year FE	yes	yes	yes	yes	yes
Observations	4844	4844	4844	4844	4844
Adjusted R-squared	0.370	0.372	0.370	0.372	0.375
F	18.97***	17.04***	16.72***	17.20***	14.68***

Note. Robust t-statistics (in parentheses) are based on the standard errors clustered by firms to address potential serial correlations in the residuals.

*** p<0.01,

** p<0.05,

* p<0.1.

Second, we used the CSR grade disclosed by the RKS (i.e., *Rank*) as an alternative measure of CSR performance and re-examined our hypotheses. RKS not only provides a social responsibility score but also ranks companies on their CSR performance from AAA+ to C (a total of 19 grades) based on this score. We constructed *Rank* by assigning a value of 19 (1) to the AAA+ (C) grade, which indicates the highest (lowest) quality of the CSR. Thus, the higher the value of *Rank*, the better the CSR performance. The results in Table 7 indicate that returnee executives with a greater geographic diversity of foreign experience have a positive effect on firms’ CSR ranking. This effect is stronger when firms have central political connections, and regional market development is better. These results are generally in line with the baseline results in Table 4.

**Table 7:** Robustness checks for an alternative measure of firms’ CSR performance.

Model No.	Model 1	Model 2	Model 3	Model 4	Model 5
Dependent variables	Rank	Rank	Rank	Rank	Rank
Geographic diversity	0.468***	0.377**	0.460***	0.359**	0.252
	(3.016)	(2.476)	(3.015)	(2.144)	(1.521)
Geographic diversity*CentralPC		0.419**			0.445**
		(2.407)			(2.133)
CentralPC		0.064			0.088
		(0.461)			(0.628)
Geographic diversity*LocalPC			0.055		-0.003
			(1.091)		(-0.054)
LocalPC			-0.021		-0.028
			(-0.591)		(-0.799)
Geographic diversity*MarketDev				0.167*	0.186*
				(1.716)	(1.924)
MarketDev				0.071	0.069
				(1.193)	(1.171)
ROA	1.013	0.963	0.995	0.957	0.915
	(1.474)	(1.411)	(1.449)	(1.388)	(1.338)
Leverage	-1.271**	-1.201**	-1.282**	-1.280**	-1.235**
	(-2.386)	(-2.269)	(-2.413)	(-2.416)	(-2.351)
Equity concentration	0.018***	0.018***	0.018***	0.018***	0.018***
	(2.899)	(2.898)	(2.873)	(2.846)	(2.831)
Firm age	-0.023	-0.021	-0.024	-0.022	-0.021
	(-1.289)	(-1.168)	(-1.294)	(-1.239)	(-1.173)
Firm size	0.929***	0.906***	0.934***	0.928***	0.913***
	(9.687)	(9.265)	(9.511)	(9.761)	(9.260)
Board size	0.047*	0.043	0.051*	0.050*	0.051*
	(1.727)	(1.574)	(1.847)	(1.887)	(1.841)
Board independence	-0.651	-0.668	-0.593	-0.605	-0.537
	(-0.785)	(-0.825)	(-0.717)	(-0.735)	(-0.666)
Duality	0.015	0.018	0.019	-0.006	-0.007
	(0.089)	(0.106)	(0.114)	(-0.038)	(-0.041)
Female executive	0.497***	0.503***	0.504***	0.499***	0.509***
	(2.975)	(3.031)	(3.020)	(3.012)	(3.100)
Executive age	0.040	0.039	0.041	0.040	0.041
	(1.492)	(1.442)	(1.546)	(1.512)	(1.539)
Foreigner	0.688**	0.696**	0.700**	0.658**	0.655**
	(2.230)	(2.276)	(2.261)	(2.118)	(2.120)
Returnee director	0.154	0.180	0.156	0.147	0.178
	(0.997)	(1.181)	(1.017)	(0.935)	(1.150)
SOE	0.327	0.321	0.330	0.334	0.332
	(1.536)	(1.511)	(1.544)	(1.565)	(1.562)
Constant	-16.304***	-15.652***	-16.439***	-16.258***	-16.036***
	(-7.722)	(-7.280)	(-7.531)	(-7.768)	(-7.345)
Industry FE	yes	yes	yes	yes	yes
Year FE	yes	yes	yes	yes	yes
Observations	4554	4554	4554	4554	4554
Adjusted R-squared	0.336	0.339	0.336	0.338	0.342
F	17.96***	16.27***	15.85***	16.24***	13.60***

Note. Robust t-statistics (in parentheses) are based on the standard errors clustered by firms to address potential serial correlations in the residuals.

*** p<0.01,

** p<0.05,

* p<0.1.

Third, considering that executives may also have work or study experience in developing regions, we also controlled for the average number of developing countries (See S1 Table for a country-specific list) where executives studied or worked abroad (*DevelopingCou*). The results in Table 8 show that returnee executives’ foreign experience in developing countries has no significant effect on CSR. This suggests that experience in developed countries is more conducive to improving firms’ CSR than experience in developing countries. Our findings remain valid after controlling for the influence of experience in developing countries.

**Table 8:** Robustness checks controlling for executives’ foreign experience in developing regions.

Model No.	Model 1	Model 2	Model 3	Model 4	Model 5
Geographic diversity	1.860***	1.542***	1.821***	1.463**	1.070*
	(3.076)	(2.632)	(3.064)	(2.249)	(1.667)
Geographic diversity*CentralPC		1.542**			1.714**
		(2.468)			(2.278)
CentralPC		0.043			0.107
		(0.081)			(0.205)
Geographic diversity*LocalPC			0.188		-0.029
			(1.006)		(-0.143)
LocalPC			-0.051		-0.066
			(-0.381)		(-0.496)
Geographic diversity*MarketDev				0.676*	0.763**
				(1.782)	(2.000)
MarketDev				0.275	0.274
				(1.187)	(1.190)
DevelopingCou	-0.141	-0.410	-0.122	-0.160	-0.483
	(-0.142)	(-0.407)	(-0.124)	(-0.161)	(-0.470)
ROA	2.386	2.340	2.322	2.208	2.193
	(0.969)	(0.956)	(0.945)	(0.894)	(0.894)
Leverage	-4.734**	-4.490**	-4.750**	-4.778**	-4.591**
	(-2.383)	(-2.278)	(-2.395)	(-2.420)	(-2.346)
Equity concentration	0.077***	0.077***	0.077***	0.076***	0.075***
	(3.240)	(3.230)	(3.221)	(3.181)	(3.162)
Firm age	-0.075	-0.067	-0.075	-0.071	-0.066
	(-1.087)	(-0.969)	(-1.073)	(-1.038)	(-0.943)
Firm size	3.602***	3.540***	3.610***	3.601***	3.555***
	(10.013)	(9.667)	(9.744)	(10.093)	(9.595)
Board size	0.169	0.161	0.180*	0.182*	0.183*
	(1.622)	(1.529)	(1.693)	(1.781)	(1.739)
Board independence	-2.667	-2.683	-2.532	-2.488	-2.312
	(-0.868)	(-0.891)	(-0.824)	(-0.818)	(-0.772)
Duality	-0.245	-0.243	-0.231	-0.316	-0.326
	(-0.390)	(-0.388)	(-0.369)	(-0.504)	(-0.524)
Female executive	2.086***	2.099***	2.110***	2.084***	2.105***
	(3.350)	(3.389)	(3.396)	(3.378)	(3.443)
Executive age	0.145	0.140	0.148	0.146	0.146
	(1.512)	(1.469)	(1.552)	(1.537)	(1.551)
Foreigner	2.961**	2.991**	3.009**	2.828**	2.823**
	(2.509)	(2.555)	(2.541)	(2.376)	(2.380)
Returnee director	0.679	0.781	0.689	0.651	0.774
	(1.162)	(1.345)	(1.181)	(1.101)	(1.318)
SOE	1.043	0.990	1.048	1.070	1.023
	(1.362)	(1.299)	(1.365)	(1.395)	(1.339)
Constant	-56.173***	-54.303***	-56.266***	-56.009***	-55.273***
	(-7.138)	(-6.791)	(-6.902)	(-7.176)	(-6.807)
Industry FE	yes	yes	yes	yes	yes
Year FE	yes	yes	yes	yes	yes
Observations	4844	4844	4844	4844	4844
Adjusted R-squared	0.370	0.373	0.370	0.373	0.376
F	17.68***	16.17***	15.77***	16.33***	14.18***

Note. Robust t-statistics (in parentheses) are based on the standard errors clustered by firms to address potential serial correlations in the residuals.

*** p<0.01,

** p<0.05,

* p<0.1.

In addition, we also tested the main hypotheses on a subsample excluding 2009 data. As we all know, China suffered a major natural disaster in 2008 (i.e., the “Wenchuan” earthquake). After that, the Chinese government issued a series of preferential policies to encourage all sectors of society to donate and help in the post-disaster reconstruction actively. The release of these policies led to a significant increase in firms’ CSR investment in 2009. Therefore, to exclude the effect of these policies, we re-tested the main hypothesis based on data covering 2010–2018, and our results remain unchanged (See Table 9).

**Table 9:** Robustness checks excluding the 2009 sample.

Model No.	Model 1	Model 2	Model 3	Model 4	Model 5
Geographic diversity	1.912***	1.583***	1.887***	1.511**	1.122*
	(3.185)	(2.702)	(3.191)	(2.292)	(1.728)
Geographic diversity*CentralPC		1.584**			1.710**
		(2.399)			(2.168)
CentralPC		0.096			0.163
		(0.177)			(0.301)
Geographic diversity*LocalPC			0.186		-0.031
			(0.947)		(-0.146)
LocalPC			-0.061		-0.078
			(-0.455)		(-0.585)
Geographic diversity*MarketDev				0.616	0.683*
				(1.587)	(1.764)
MarketDev				0.271	0.266
				(1.160)	(1.146)
ROA	3.041	2.894	2.975	2.820	2.706
	(1.168)	(1.119)	(1.144)	(1.079)	(1.043)
Leverage	-4.907**	-4.682**	-4.935**	-4.935**	-4.783**
	(-2.377)	(-2.283)	(-2.396)	(-2.404)	(-2.349)
Equity concentration	0.074***	0.074***	0.074***	0.074***	0.073***
	(3.055)	(3.054)	(3.031)	(2.996)	(2.983)
Firm age	-0.088	-0.080	-0.089	-0.084	-0.080
	(-1.268)	(-1.155)	(-1.263)	(-1.218)	(-1.143)
Firm size	3.670***	3.604***	3.683***	3.667***	3.624***
	(9.932)	(9.553)	(9.719)	(10.005)	(9.514)
Board size	0.175*	0.165	0.188*	0.188*	0.189*
	(1.673)	(1.556)	(1.758)	(1.829)	(1.776)
Board independence	-2.441	-2.457	-2.273	-2.261	-2.034
	(-0.779)	(-0.801)	(-0.727)	(-0.728)	(-0.667)
Duality	-0.199	-0.183	-0.184	-0.281	-0.277
	(-0.307)	(-0.283)	(-0.285)	(-0.434)	(-0.431)
Female executive	2.068***	2.090***	2.090***	2.075***	2.107***
	(3.247)	(3.299)	(3.287)	(3.284)	(3.361)
Executive age	0.157	0.151	0.161	0.157	0.157
	(1.547)	(1.505)	(1.592)	(1.561)	(1.580)
Foreigner	3.044**	3.060**	3.087**	2.930**	2.908**
	(2.491)	(2.526)	(2.518)	(2.380)	(2.370)
Returnee director	0.682	0.778	0.690	0.651	0.766
	(1.134)	(1.306)	(1.150)	(1.069)	(1.269)
SOE	1.218	1.176	1.225	1.245	1.213
	(1.539)	(1.491)	(1.544)	(1.568)	(1.534)
Constant	-57.773***	-55.790***	-58.037***	-57.536***	-56.900***
	(-7.185)	(-6.790)	(-6.975)	(-7.210)	(-6.818)
Industry FE	yes	yes	yes	yes	yes
Year FE	yes	yes	yes	yes	yes
Observations	4554	4554	4554	4554	4554
Adjusted R-squared	0.346	0.349	0.346	0.349	0.352
F	19.55***	17.74***	17.25***	17.75***	14.91***

Note. Robust t-statistics (in parentheses) are based on the standard errors clustered by firms to address potential serial correlations in the residuals.

*** p<0.01,

** p<0.05,

* p<0.1.

### Heterogeneity analysis

#### Foreign working experience vs. foreign education experience

Different experiences or competencies of managers affect managerial decision-making in different ways. Prior studies suggest that international work experience is more helpful for returnees to disseminate advanced knowledge of business and corporate governance in emerging market firms [81, 82]. Therefore, we predict that returnee executives with foreign work experience may significantly influence CSR more than those with foreign education experience. We created two variables (i.e., *Geographic diversity_E* and *Geographic diversity_W*) to measure the education and work experiences of returnee executives from developed countries. *Geographic diversity_E* is the average number of developed countries where returnee executives studied abroad. *Geographic diversity_W* is the average number of developed countries where returnee executives worked abroad. In addition, we similarly constructed two variables (i.e., *DevelopingCou_E* and *DevelopingCou_W*) to measure the education and work experience obtained by returnee executives in developing countries. *DevelopingCou_E* is the average number of developing countries where returnee executives studied abroad. *DevelopingCou_W* is the average number of developing countries where returnee executives worked abroad. Models 1 and 2 in Table 10 indicate that foreign work experience from developed countries may be more helpful to returnee executives in improving firms’ CSR than foreign education experience gained from developed countries. Furthermore, we find that neither education nor work experience in developing countries has a significant impact on firms’ CSR.

**Table 10:** Heterogeneity analysis: Different types of foreign experience and industry attributes.

Model No.	Model 1	Model 2	Model 3	Model 4
	Foreign education experience	Foreign working experience	Heavy-polluted	Light-polluted
Geographic diversity			0.397	2.632***
			(0.442)	(3.572)
Geographic diversity_E	1.120			
	(1.158)			
DevelopingCou_E	0.132			
	(0.037)			
Geographic diversity_W		2.364***		
		(3.285)		
DevelopingCou_W		-0.252		
		(-0.249)		
ROA	2.088	2.343	1.395	2.910
	(0.845)	(0.947)	(0.306)	(1.020)
Leverage	-4.907**	-4.746**	-6.101*	-4.155*
	(-2.440)	(-2.386)	(-1.779)	(-1.708)
Equity concentration	0.075***	0.078***	0.125***	0.059**
	(3.133)	(3.291)	(3.110)	(2.036)
Firm age	-0.080	-0.079	-0.111	-0.060
	(-1.152)	(-1.147)	(-0.803)	(-0.773)
Firm size	3.716***	3.582***	3.601***	3.539***
	(10.096)	(9.876)	(5.677)	(8.263)
Board size	0.171	0.162	0.250	0.100
	(1.639)	(1.553)	(1.164)	(0.879)
Board independence	-2.815	-2.720	0.976	-3.552
	(-0.902)	(-0.891)	(0.176)	(-0.977)
Duality	-0.232	-0.228	-1.058	-0.037
	(-0.364)	(-0.363)	(-0.953)	(-0.048)
Female executive	2.176***	2.128***	1.816*	2.055***
	(3.477)	(3.431)	(1.761)	(2.670)
Executive age	0.144	0.149	0.116	0.132
	(1.497)	(1.569)	(0.693)	(1.111)
Foreigner	3.185***	2.902**	3.449	2.771**
	(2.648)	(2.484)	(1.383)	(2.085)
Returnee director	0.913	0.782	0.464	1.000
	(1.560)	(1.347)	(0.408)	(1.463)
SOE	0.965	1.054	1.667	0.936
	(1.240)	(1.386)	(1.202)	(1.008)
Constant	-58.334***	-55.883***	-58.414***	-52.854***
	(-7.293)	(-7.043)	(-4.320)	(-5.585)
Industry FE	yes	yes	yes	yes
Year FE	yes	yes	yes	yes
Observations	4844	4844	1388	3456
Adjusted R-squared	0.365	0.372	0.415	0.361
F	16.85***	18.01***	6.36***	14.64***

Note. Robust t-statistics (in parentheses) are based on the standard errors clustered by firms to address potential serial correlations in the residuals.

*** p<0.01,

** p<0.05,

* p<0.1.

#### Heavily polluting industries vs. lightly polluting industries

The impact of industry is of great importance, as different industries may have evolved varying approaches to CSR [83]. Previous studies have found a systematic relationship between broad industry characteristics and CSR activities. A recent study [84] documents the positive impact of executives’ overseas backgrounds on the green innovation performance of firms. It highlights that this effect differs significantly between firms in different industries (e.g., heavily and lightly polluting industries). Firms in heavily polluting industries lead to high levels of air and environmental pollution, while firms in lightly polluting industries do not. Thus, based on the Guidelines for Environmental Information Disclosure of Listed Companies issued by China’s Ministry of Environmental Protection in 2010, we divided the sample firms into two groups: those in heavily polluting industries and those in lightly polluting industries (See S3 Table for details), in order to examine the heterogeneous effect of the industry. Models 3 and 4 of Table 10 report the results for firms in heavily polluting industries and lightly polluting industries, respectively. The results show that returnee executives’ geographic diversity promotes the CSR of firms in lightly polluting industries but not heavily polluting industries. This may be because there is a self-selection effect for returnee executives who choose to work in heavily polluting industries. That is, only returnee executives who do not value social responsibility are willing to work in highly polluting industries with poor social responsibility performance.

### Mechanism analysis

#### The mediating effect of corporate donation

Previous research shows that having returnees on the corporate board remarkably boosts firms’ donations [85]. Thus, we predict that returnee executives can improve CSR by promoting corporate giving. Referring to prior studies [86, 87], we measure corporate donation as the natural logarithm of the firm’s donation amount plus 1. We test for the mediating role of corporate donation using the Sobel intermediary factor test method [88]. The indirect effect is how the independent variable transmits its effect on the dependent variable via the mediator [89]. The Sobel test verifies the significance of the indirect effect. Table 11 reports the results of the mediating role of corporate donation. The Sobel test verifies the mediating effect of *Dona* [Sobel = 2.66 (0.06), p < 0.01] on the relationship between returnee executives’ geographic diversity and firms’ CSR. In addition, we used the bootstrap method [90, 91] to re-examine the mediating role of corporate donations, as some scholars have recently raised doubts about the Sobel test. 5000 samples were selected using the nonparametric percentile method for deviation correction, with a confidence interval (CI) of 95%. We find that the 95% CI ranges from 0.02 to 0.28. These results further verify the mediating role of corporate donations.

**Table 11:** Mechanism analysis.

Model No.	Model 1	Model 2	Model 3	Model 4
	Corporate donation		Corporate green innovation	
Dependent variables	Dona	CSR_Score	GreenInn	CSR_Score
Dona		0.825***		
		(14.850)		
Geographic diversity	0.186***	1.692***	0.111***	1.723***
	(2.700)	(6.390)	(4.070)	(6.400)
GreenInn				1.188***
				(8.330)
ROA	2.764***	0.091	0.861***	1.298
	(7.160)	(0.060)	(5.630)	(0.860)
Leverage	-1.109***	-3.834***	0.059	-4.853***
	(-4.650)	(-4.170)	(0.630)	(-5.200)
Equity concentration	0.004	0.074***	-0.004***	0.082***
	(1.370)	(7.500)	(-4.160)	(8.190)
Firm age	-0.032***	-0.049*	-0.019***	-0.051*
	(-4.180)	(-1.660)	(-6.120)	(-1.690)
Firm size	0.687***	3.036***	0.316***	3.232***
	(18.820)	(20.850)	(21.890)	(21.590)
Board size	0.013	0.158***	0.009*	0.159***
	(0.940)	(2.980)	(1.710)	(2.960)
Board independence	0.757	-3.285*	-0.384*	-2.230
	(1.510)	(-1.700)	(-1.930)	(-1.140)
Duality	-0.058	-0.192	0.125***	-0.425
	(-0.600)	(-0.510)	(3.230)	(-1.110)
Female executive	0.053	2.046***	0.143***	1.911***
	(0.710)	(7.100)	(4.810)	(6.510)
Executive age	0.023*	0.126***	-0.007	0.153***
	(1.940)	(2.740)	(-1.410)	(3.280)
Foreigner	0.223	2.772***	0.198***	2.749***
	(1.500)	(4.860)	(3.370)	(4.730)
Returnee director	-0.113	0.769**	-0.012	0.673**
	(-1.450)	(2.570)	(-0.400)	(2.220)
SOE	-0.559***	1.506***	0.047	0.991***
	(-6.070)	(4.230)	(1.300)	(2.750)
Constant	-13.410***	-55.410***	-6.441***	-58.910***
	(-15.340)	(-16.080)	(-18.610)	(-16.640)
Industry FE	yes	yes	yes	yes
Year FE	yes	yes	yes	yes
Observations	4844	4844	4839	4839
Adjusted R-squared	0.196	0.398	0.260	0.379
F	31.97***	82.93***	45.78***	76.75***

Note. Robust t-statistics (in parentheses) are based on the standard errors clustered by firms to address potential serial correlations in the residuals.

*** p<0.01,

** p<0.05,

* p<0.1.

#### The mediating effect of corporate green innovation

Previous literature suggests that returnee executives can promote corporate green innovation [21, 84], and the increased level of green innovation contributes to CSR improvement. Therefore, we examined whether returnee executives’ geographic diversity influences firms’ CSR performance through corporate green innovation. Specifically, we select the natural logarithm of the number of green patent applications of firms plus 1 to measure firms’ green innovation (*GreenInn*), which is widely adopted in the literature [84, 92, 93]. Models 3 and 4 of Table 11 exhibit the results of the mediating role of corporate green innovation. The results of the Sobel test verify the mediating effect of *GreenInn* [Sobel = 3.65 (0.04), p < 0.01] on the relationship between returnee executives’ geographic diversity and CSR. In addition, the results of the Bootstrap method indicate that the indirect effect of the geographic diversity of foreign experience on firms’ CSR through *GreenInn* is significant, as its CI ranges from 0.04 to 0.22.

## Discussion

This paper provides a theoretical analysis and relevant empirical evidence on the effects of returnee executives’ geographic diversity on firms’ CSR performance to illustrate the effect of returnees on corporate social behavior.

First, the hypothesis concerning the positive impact of the geographic diversity of returnee executives’ foreign experience on CSR (H1) is verified, which expands the literature on the diversity of foreign experience to some extent. Prior studies show that the geographic diversity of executives’ foreign experience differentially affects firm outcomes in creative innovation [22], post-acquisition performance [23], and internationalization [24]. This paper extends the existing literature by demonstrating the positive effect of the geographic diversity of returnee executives’ foreign experience on firms’ CSR performance.

Second, this paper analyzes the two boundary conditions of the geographic diversity of foreign experience and CSR. One is corporate political connections (H2), measured by the number of TMT and board members who have worked in central-level and local-level government agencies, respectively. Previous studies [52, 72] suggest that political connections at different levels may affect firms’ social behavior differently, given China’s institutional complexity. This paper further demonstrates that central and local government political connections differentially moderate the relationship between the geographic diversity of executives’ foreign experience and CSR. More specifically, political connections at the central level significantly strengthen the positive effect of returnee executives’ geographic diversity on firms’ CSR, while political connections at the local level do not. This discrepancy in results may be ascribed to China’s unique political structure. Under this political structure, central and local government officials develop different mindsets and goals [73, 74]. In particular, local government officials are generally perceived to prioritize local economic growth over CSR improvement, influenced by both China’s regionally decentralized authoritarian regime [94] and political promotion system [95]. In this sense, executives who have worked in local government agencies are well aware of this and are likely to communicate this information to the companies they serve. In doing so, firms’ CSR investments may be constrained. This may be why we do not observe a significant moderating effect of local political connections on the relationship between returnee executives and CSR.

However, unlike local governments, which see economic growth as their primary goal, the central government is more concerned with social and environmental outcomes [71], especially since 2006, when the central government initiated a new goal of developing a “harmonious society.” In this vein, executives associated with the central government convey the urgency and priority of CSR to other members of the TMT, such as returnee executives, because the harmonious society policy is seen to share common goals with CSR [96]. As a result, central-level political connections notably intensify the influence of returnee executives on CSR, while local-level political connections do not.

Another boundary condition is regional market development (H3), a key factor impacting firms’ CSR [97–99]. Literature states that in regions with high levels of market development, firms have fewer external constraints and more freedom of action, and their executives’ managerial discretion is also greater [59, 60]. This paper reveals that good market development increases the discretion of returnee executives in CSR decisions, thereby augmenting the positive effect of the geographic diversity of returnee executives’ foreign experience on firms’ CSR.

## Conclusion

A large body of literature concentrates on the firm- and institution-level factors driving CSR, with less attention paid to the impact of returnee executives. This paper investigates the impact of returnee executives (measured by the geographic diversity of foreign experience) on firms’ CSR based on China’s A-share-listed companies for the period 2009–2018 and draws the following conclusions: (1) the geographic diversity of returnee executives’ foreign experience positively affects firms’ CSR; (2) this effect is stronger in firms with political connections with the central government; (3) this effect is stronger in regions with good market development. In addition, the mechanism analysis shows that TMTs with a broad scope of foreign experience drive firms’ CSR by promoting corporate donations and green innovation.

### Theoretical contribution

This paper contributes to the existing literature in several ways. First, this study adds to the research on CSR determinants in China. Scholarly work on CSR has demonstrated that a firm’s CSR level is determined by a combination of many factors, including institutional-level factors [70, 100, 101], enterprises’ characteristics [9, 53, 102], corporate governance structure [103, 104], executives’ experience and values [8, 105, 106], and financial performance [107, 108]. However, little attention has been paid to returnees. In contrast to previous studies [12, 13, 109] focusing on the impact of the number of returnee executives or the duration of foreign experience on CSR, this paper documents the positive effect of the geographic diversity of returnee executives’ foreign experience on firms’ CSR. The results of this paper enrich our understanding of the CSR determinants in emerging markets.

This study also extends the work on the role of returnees in China. Previous studies suggest that returnees can influence the behavior and performance of firms, including new venture creation [1–3], innovation performance [5, 21], internationalization [4], fraudulent behavior [82], tax avoidance [110], and cross-border acquisitions [111]. In contrast to their primary focus on the number of returnees or the length of their stay abroad, this paper concentrates on the impact of returnees’ heterogeneity in geographical scope on firm behavior, revealing the positive effect of returnee executives’ geographic diversity on CSR. Our findings suggest that the geographic diversity of foreign experience may be an essential indicator for exploring the impact of returnees on firm behavior.

This study adds to understanding the boundary conditions of international knowledge transfer. Although existing literature has identified returnee executives as an important channel of knowledge transfer for emerging market firms, there is limited understanding of the boundary conditions of such knowledge transfer. Previous research shows certain factors that may hinder international knowledge transfer by returnee executives, such as difficult interpersonal relationships with colleagues and the administrative heritage of the firm [112]. This paper indicates that certain organizational (i.e., corporate political connections with the central government) and institutional (i.e., good market development) factors can facilitate knowledge transfer by investigating the boundary conditions for the relationship between returnee executives’ geographic diversity and firms’ CSR. Finally, this paper contributes to the literature on how returnees influence corporate social behavior by demonstrating the mediating role of corporate donations and green innovation on the relationship between returnee executives and CSR.

### Practical implications

This paper contributes to understanding returnees in China by demonstrating the positive effect of the geographic diversity of returnee executives on firms’ CSR behavior. The research in this paper may have managerial implications in the following aspects. First, when hiring returnees as executives, companies should give preference to those with foreign experience in multiple countries. Second, hiring people who have worked in central government agencies as executives can help returnee executives play a more significant role in improving CSR. Third, accelerating the process of regional marketization and guaranteeing firms’ managerial discretion in operating activities can promote the knowledge transfer of returnee executives. Finally, our findings are relevant not only to China but also to other emerging economies, especially those with weak institutional systems.

### Limitations and future research

Our study has several limitations. First, other characteristics of returnee executives’ foreign experience may influence the relationship between the geographic diversity of foreign experience and CSR performance. Our study primarily captures the effect of the breadth of foreign experience on CSR. Future research could incorporate the length of time that returnee executives spend abroad or the heterogeneity in the country of origin of their overseas experience into the theoretical framework to comprehensively examine the impact of overseas experience on CSR.

Moreover, this study concentrates on the impact of the diversity of TMT’s foreign experience on CSR, as TMT is the information processing and decision-making center of the firm. However, the foreign experience of the board of directors is also widespread; in particular, the board’s primary role is to advise and supervise TMT members [113], which has been found to enhance the social performance of firms [85]. Thus, future research should explore the joint impact of returnee executives and directors on CSR. For instance, how and when does CEO duality (i.e., a firm’s CEO and chairman are the same person) affect corporate social performance, which leads to different firm performance [114]?

Furthermore, this study focuses on the overall performance of CSR. The findings of this paper are not necessarily applicable to other social initiatives. Future research could investigate the direct and indirect effects of the breadth and width of executives’ experiences abroad on, for example, corporate philanthropy [115], energy innovation [116], and environmental performance [117].

In addition, our sample is limited to Chinese-listed companies that voluntarily disclose CSR reports and are included in the RKS. While this focus allowed us to obtain authentic and reliable data on CSR performance, thus ensuring the credibility of our findings, our results may not be generalizable to other firms that are not listed or do not explicitly disclose their CSR reports. In addition, our study is based on the Chinese context. Although China is one of the largest emerging economies in the world, our findings may not be generalizable to other emerging economies. Therefore, a fruitful direction for future research would be to investigate whether our findings hold true for firms that do not explicitly disclose CSR reports and whether they can be replicated in other emerging economies.

## Supporting information

S1 Table Descriptive statistics of the executive-level data. (DOCX)

S2 Table MCTI rating system developed by RKS. (DOCX)

S3 Table Industry distribution of sample firms. (DOCX)

S1 Data Research data. (ZIP)
