# Supplementary material for: Diversity of returnee executives’ foreign experience and corporate social responsibility performance
Source: PLoS One. 2024 Apr 1;19(4):e0300262. doi: 10.1371/journal.pone.0300262 (PMC10984457; doi:10.1371/journal.pone.0300262)
Supplement: S1 Table — (DOCX) [file pone.0300262.s001.docx]

# S1 Table

Approximately 4.45% of the observations (1733 executive-firm-year observations) have foreign experience; of these, 1546 executive-firm-year observations have foreign experience in developed countries, 428 executive-firm-year observations have that in developing countries, 241 executive-firm-year observations have foreign experience in both developed and developing countries. Besides, 860 executive-firm-year observations have foreign study experience; of these, 160 executive-firm-year observations correspond to short-time visits, training, and post-docs in foreign academic institutes, 125 executive-firm-year observations correspond to foreign bachelor degrees, 532 executive-firm-year observations correspond to foreign master degrees, 143 executive-firm-year observations correspond to foreign doctoral degrees. Additionally, 1125 executive-firm-year observations have foreign work experience; of these, 936 executive-firm-year observations have worked in developed countries, and 1009 executive-firm-year observations have served as middle or senior managers in foreign companies.

Most of the executives received their foreign study experience in the United States (375 executive-firm-year observations), followed by the United Kingdom (193 executive-firm-year observations), Australia (64), France (59), and Japan (44). Most executives get foreign work experience in the United States (394 executive-firm-year observations), followed by Hong Kong (189), Japan (98), Singapore (86), and Canada (75). In addition, we also count other characteristics concerning executives generally reported in the literature. See the table below for details.

**Table I. Summary statistics of the executive-level data.**

| **Variables** | **Mean** | **Median** | **SD** | **N** |
| --- | --- | --- | --- | --- |
| Nationality | 0.012 | 0 | 0.109 | 38983 |
| Female executive | 0.118 | 0 | 0.322 | 38983 |
| Executive age | 47.727 | 48 | 6.428 | 38983 |
| Director | 0.279 | 0 | 0.449 | 38983 |
| IsDuality | 0.022 | 0 | 0.148 | 38983 |
| Tenure | 4.28 | 3 | 3.263 | 38983 |
| Foreign experience | 0.044 | 0 | 0.206 | 38983 |
| Developed | 0.04 | 0 | 0.195 | 38983 |
| Developing | 0.011 | 0 | 0.104 | 38983 |
| Foreign study experience | 0.022 | 0 | 0.147 | 38983 |
| Study_Developed | 0.022 | 0 | 0.146 | 38983 |
| Study_Developing | 0.001 | 0 | 0.023 | 38983 |
| Foreign bachelor degree | 0.003 | 0 | 0.057 | 38983 |
| Foreign master degree | 0.014 | 0 | 0.116 | 38983 |
| Foreign doctoral degree | 0.004 | 0 | 0.06 | 38983 |
| Foreign visiting scholar/training/postdoc | 0.004 | 0 | 0.064 | 38983 |
| Social science | 0.015 | 0 | 0.123 | 38983 |
| Foreign work experience | 0.029 | 0 | 0.167 | 38983 |
| Work_Developed | 0.024 | 0 | 0.153 | 38983 |
| Work_Developing | 0.011 | 0 | 0.102 | 38983 |
| Middle/senior manager | 0.026 | 0 | 0.159 | 38983 |
| Foreign study and work experience | 0.006 | 0 | 0.08 | 38983 |

Note. Table I summarizes the descriptive statistics of the executive-firm-year data set from 2009 to 2018. *Nationality* is a dummy equal to one if an executive is a foreign national. *Female executive* is a dummy equal to one if an executive is female. *Executive age* is the difference between the current year and the birth year of the executive. *Director* is a dummy equal to one if an executive is a member of the board of directors. *IsDuality* is a dummy equal to one if the executive serves as both Chairman and CEO. *Tenure* is one plus the difference between the current year and the year when the individual joined the TMT of a given firm. *Foreign experience* is a dummy equal to one if an executive has foreign study or foreign work experience. *Developed* is a dummy equal to one if an executive has foreign study or foreign work experience in developed countries. *Developing* is a dummy equal to one if an executive has foreign study or work experience in developing countries. *Foreign study experience* is a dummy equal to one if an executive has foreign study experience comprising education, training, visiting, or post-doc. *Study_Developed* is a dummy equal to one if an executive has foreign study experience in developed countries. *Study_Developing* is a dummy equal to one if an executive has foreign study experience in developing countries. *Foreign bachelor degree* is a dummy equal to one if an executive holds a bachelor degree from a foreign country. *Foreign master degree* is a dummy equal to one if an executive holds a master degree from a foreign country. *Foreign doctoral degree* is a dummy equal to one if an executive holds a doctoral degree from a foreign country. *Foreign visiting scholar/training/postdoc* is a dummy equal to one if an executive was a visiting scholar or post-doc or did a short-term training program. *Social science* is a dummy equal to one if an executive has majored in social sciences in a foreign country. *Foreign work experience* is a dummy equal to one if an executive has foreign work experience. *Work_Developed* is a dummy equal to one if an executive has foreign work experience in developed countries. *Work_Developing* is a dummy equal to one if an executive has foreign work experience in developing countries. *Middle/senior manager* is a dummy equal to one if an executive holds middle-level or higher management positions in foreign work experience. *Foreign study and work experience* is a dummy equal to one if an executive has both foreign study experience and foreign work experience.

**Table II. Developed and developing countries/regions covered by the study.**

| **Developed countries/regions** | **Developing countries/regions** |
| --- | --- |
| Australia | Bangladesh |
| Austria | Brazil |
| Belgium | Cambodia |
| Canada | Congo |
| Finland | India |
| France | Indonesia |
| Germany | Iran |
| Hong Kong, China | Kazakhstan |
| Ireland | Kenya |
| Italy | Laos |
| Japan | Libya |
| Luxembourg | Malaysia |
| Macao, China | Mexico |
| Netherlands | Mongolia |
| New Zealand | Myanmar |
| Norway | Nigeria |
| Poland | Pakistan |
| Singapore | Philippines |
| South Korea | Qatar |
| Spain | Russia |
| Sweden | Senegal |
| Switzerland | Thailand |
| Taiwan, China | Tunisia |
| United Kingdom | Türkiye |
| United States of America | Turkmenistan |
|  | Uganda |
|  | United Arab Emirates |
|  | Venezuela |
|  | Vietnam |
|  | Zimbabwe |
