# Supplementary material for: Diversity of returnee executives’ foreign experience and corporate social responsibility performance
Source: PLoS One. 2024 Apr 1;19(4):e0300262. doi: 10.1371/journal.pone.0300262 (PMC10984457; doi:10.1371/journal.pone.0300262)
Supplement: S2 Table — (DOCX) [file pone.0300262.s002.docx]

# S2 Table

RKS is completely independent of the firms it rates. The CSR rating system developed by RKS builds on the Global Reporting Initiative (3.0) framework and integrates CSR elements specific to China. Its original evaluation data comes from companies’ publicly available social responsibility reports, official websites, and news media. Specifically, RKS sets 15 primary and 63 secondary indicators based on the MCTI evaluation system to comprehensively evaluate the firm’s CSR performance level (See the following table). The rating system uses a structured expert scoring method with a total score of 100 points. The higher the social responsibility score in the RKS, the better the firm’s CSR performance.

**Table III. MCTI rating system developed by RKS.**

| **Four evaluation dimensions** | | | **First-level sub-indicators** | **Second-level sub-indicators** |
| --- | --- | --- | --- | --- |
| **Dimension** | **Score** | **Weight** |  |  |
| Macrocosm (M) | 30 | 30% | M1 Strategy | M1.1 Overall responsibility strategy information  M1.2 Information on sustainable development adaptation and response  M1.3 Responsibility strategy and valid firm matching information  M1.4 Information on the consideration of social responsibility by corporate executives at the strategic level  M1.5 Social responsibility goal setting and achievement information |
|  |  |  | M2 Governance | M2.1 Basic information about the company  M2.2 Values, principles, and guidelines information  M2.3 Social responsibility management organization information  M2.4 Information about decision-making processes and structures  M2.5 Governance transparency information  M2.6 Risk management information  M2.7 Business ethics governance information  M2.8 Internal practice information |
|  |  |  | M3 Stakeholder | M3.1 Stakeholder definition and identification information  M3.2 Stakeholder communication information  M3.3 Stakeholder opinion information |
| Content (C) | 45 | 45% | C1 Economic performance | C1.1 Profit and return information  C1.2 Year-over-year economic information  C1.3 Basic information on major products or services |
|  |  |  | C2 Labor and human rights | C2.1 Information on employment and employment relations  C2.2 Employee career development information  C2.3 Occupational health and safety information  C2.4 Information on human rights protection  C2.5 Working conditions and social security information  C2.6 Information on social dialogue and care  C2.7 Responsible education information |
|  |  |  | C3 Environment | C3.1 Overall environmental management information  C3.2 Pollution prevention information  C3.3 Sustainable resource use information  C3.4 Climate change mitigation and adaptation information |
|  |  |  | C4 Fair operation | C4.1 Anti-corruption management information  C4.2 Promotion of social responsibility information within the sphere of influence |
|  |  |  | C5 Consumer | C5.1 Providing information on quality assurance of products or services  C5.2 Consumer (customer) management information  C5.3 Protection of consumer safety and health information  C5.4 Consumer (customer) service information  C5.5 Protection of consumer (customer) data and privacy information  C5.6 Consumer education information |
|  |  |  | C6 Community engagement and development | C6.1 Information on public welfare donation  C6.2 Volunteerism information  C6.3 Political participation information  C6.4 Job creation information  C6.5 Information on scientific and technological development  C6.6 Information on wealth creation and income  C6.7 Information on health promotion  C6.8 Social investment information |
| Technique (T) | 15 | 15% | T1 Content of the balance | T1.1 Completeness  T1.2 Pertinence |
|  |  |  | T2 Information comparability | T2.1 Consistency  T2.2 Data |
|  |  |  | T3 Report innovation | T3.1 Innovativeness  T3.2 Effectiveness of innovation |
|  |  |  | T4 Credibility and  transparency | T4.1 Degree of disclosure of stakeholders’ opinions  T4.2 Degree of third-party validation (comprehensiveness, depth, principle, none)  T4.3 Authority of third-party validation institutions  T4.4 Effectiveness of the feedback mechanism of the report readers’ opinions and suggestions |
|  |  |  | T5 Normative | T5.1 Reporting on policy compliance  T5.2 Reporting standards  T5.3 Report seriousness |
|  |  |  | T6 Availability and effectiveness of information delivery | T6.1 Report language version adequacy  T6.2 Access to reports and particular ways to access reports for people with special needs  T6.3 Report on the improvement of the art design and typesetting to the disclosure effect  T6.4 Degree of graphical presentation of report data and information |
| Industry (I) | 10 | 10% | **Industry** | **Number of level-2 sub-indexes** |
|  |  |  | Extractive industries | 5 |
|  |  |  | Communication and cultural industries | 2 |
|  |  |  | Production and supply of electricity, gas, and water | 5 |
|  |  |  | Electronics industry | 5 |
|  |  |  | Real estate industry | 13 |
|  |  |  | Textile, clothing, fur manufacturing | 5 |
|  |  |  | Machinery, equipment, instrumentation manufacturing | 6 |
|  |  |  | Construction industry | 10 |
|  |  |  | Financial insurance industry | 6 |
|  |  |  | Transportation and warehousing industry | 7 |
|  |  |  | Metal and non-metal manufacturing | 13 |
|  |  |  | Wood furniture manufacturing | 8 |
|  |  |  | Agriculture, forestry, animal husbandry, and fishery | 7 |
|  |  |  | Wholesale and retail trade | 6 |
|  |  |  | Social services | 4 |
|  |  |  | Petroleum, chemical, and plastic industries | 11 |
|  |  |  | Food and beverage industry | 6 |
|  |  |  | Information technology industry | 3 |
|  |  |  | Pharmaceutical, biological products industry | 14 |
|  |  |  | Pulp and paper industry | 5 |
|  |  |  | Other manufacturing | 0 |
|  |  |  | Comprehensive | 0 |

Note: (1) The sub-indicator systems of the macrocosm (M), content (C), and technology (T) contain 15 first-level sub-indicators and 63 second-level sub-indicators. The sub-indicator system of industry (I) contains 22 industries and a total of 141 indicators. Among them, firms in other manufacturing and comprehensive industries do not obtain an industry (I) score; instead, they obtain higher weights in C and T.

(2) Due to space limitations, the table no longer lists the industry sub-indicators and only lists the number of sub-indicators of each industry.
