# Supplementary material for: Diversity of returnee executives’ foreign experience and corporate social responsibility performance
Source: PLoS One. 2024 Apr 1;19(4):e0300262. doi: 10.1371/journal.pone.0300262 (PMC10984457; doi:10.1371/journal.pone.0300262)
Supplement: S3 Table — (DOCX) [file pone.0300262.s003.docx]

# S3 Table

**Table IV. Industry distribution of sample firms.**

| Industry | N | Percentage |
| --- | --- | --- |
| Agriculture (A) | 62 | 1.28% |
| Mining (B) | 237 | 4.89% |
| Manufacturing (C) |  |  |
| Food, beverage, textile, garment and leather | 326 | 6.73% |
| Papermaking, furniture, medical and chemical | 742 | 15.32% |
| Metal, nonmetal, machinery and electronics | 1652 | 34.10% |
| Other manufacturing | 17 | 0.35% |
| Electronic and gas (D) | 285 | 5.88% |
| Construction (E) | 155 | 3.20% |
| Wholesale and retail (F) | 267 | 5.51% |
| Transportation (G) | 305 | 6.30% |
| Accommodation and catering (H) | 10 | 0.21% |
| Information and technology (I) | 240 | 4.95% |
| Real estate (K) | 335 | 6.92% |
| Leasing and business services (L) | 54 | 1.11% |
| Scientific research and technical services (M) | 9 | 0.19% |
| Public facilities management (N) | 31 | 0.64% |
| Health and social work (Q) | 30 | 0.62% |
| Culture, sports and entertainment (R) | 50 | 1.03% |
| Comprehensive (S) | 37 | 0.76% |
| Total | 4844 |  |

Note. While all industries have firms with returnee executives, the industries in which more firms do so are manufacturing, information and technology, mining, transportation, construction, real estate, and wholesale and retail. Not surprisingly, scientific knowledge acquired abroad may play a crucial role in these industries.

Following the Guidelines for Environmental Information Disclosure of Listed Companies issued by China’s Ministry of Environmental Protection in 2010, heavily polluting industries include coal-fired power plants, iron and steel, cement, electrolytic aluminum, coal, chemicals, petrochemicals, building materials, paper, brewing, pharmaceuticals, fermentation, textiles, tanning, and mining. In our study, heavily polluting industries include 1388 firm-year observations, and lightly polluting industries include 3456 firm-year observations. See the table below for details.

**Table V. Sample distribution by heavily and lightly polluting industries.**

| **Industry** | **Industry code** | **Industry name** | **N** | **Total** |
| --- | --- | --- | --- | --- |
| **Heavy-polluted industry** | B06 | Mining and washing of coal | 92 | 1388 |
|  | B07 | Oil and gas extraction | 34 |  |
|  | B08 | Ferrous metal ore mining | 18 |  |
|  | B09 | Nonferrous metal mining | 70 |  |
|  | C15 | Beverage production | 115 |  |
|  | C17 | Textile manufacturing | 53 |  |
|  | C18 | Apparel manufacturing | 42 |  |
|  | C19 | Leather, fur, and feather production | 15 |  |
|  | C22 | Paper production | 44 |  |
|  | C25 | Petroleum processing | 40 |  |
|  | C26 | Chemical materials manufacturing | 250 |  |
|  | C28 | Chemical fiber manufacturing | 35 |  |
|  | C29 | Rubber and plastic production | 53 |  |
|  | C30 | Nonmetallic mineral production | 127 |  |
|  | C31 | Ferrous metal smelting | 131 |  |
|  | C32 | Nonferrous metal smelting | 221 |  |
|  | C33 | Metal product manufacturing | 48 |  |
| **Light-polluted industry** | A01 | Agriculture | 20 | 3456 |
|  | A02 | Forestry | 7 |  |
|  | A03 | Animal husbandry | 20 |  |
|  | A04 | Fishery | 15 |  |
|  | B11 | Mining support industry | 23 |  |
|  | C13 | Agricultural processing | 58 |  |
|  | C14 | Food production | 43 |  |
|  | C21 | Furniture manufacturing | 10 |  |
|  | C23 | Printing and recording | 24 |  |
|  | C24 | Cultural manufacturing | 8 |  |
|  | C27 | Pharmaceutical manufacturing | 278 |  |
|  | C34 | General equipment manufacturing | 141 |  |
|  | C35 | Special equipment manufacturing | 198 |  |
|  | C36 | Automotive manufacturing | 143 |  |
|  | C37 | Transportation equipment | 60 |  |
|  | C38 | Electrical machinery and equipment | 207 |  |
|  | C39 | Computers and communications equipment | 348 |  |
|  | C40 | Instrument manufacturing | 28 |  |
|  | C41 | Other manufacturing | 9 |  |
|  | C42 | Waste utilization industry | 8 |  |
|  | D44 | Production and supply of electric power and heat power | 228 |  |
|  | D45 | Production and Distribution of Gas | 29 |  |
|  | D46 | Water manufacturing and supply | 28 |  |
|  | E48 | Civil engineering construction | 131 |  |
|  | E50 | Architectural decoration and other construction industry | 24 |  |
|  | F51 | Wholesale trade | 147 |  |
|  | F52 | Retail trade | 120 |  |
|  | G53 | Railway transportation | 26 |  |
|  | G54 | Land transportation | 120 |  |
|  | G55 | Ship transportation | 101 |  |
|  | G56 | Flight transportation | 39 |  |
|  | G58 | Loading and transportation agency | 7 |  |
|  | G59 | Storage | 10 |  |
|  | G60 | Post services | 2 |  |
|  | H61 | Accommodation | 10 |  |
|  | I63 | Telecommunications radio and television, and satellite transmission services | 39 |  |
|  | I64 | Internet and related services | 51 |  |
|  | I65 | Software and information technology service | 150 |  |
|  | K70 | Real estate | 335 |  |
|  | L72 | Business services | 54 |  |
|  | M73 | Research and experimental development | 2 |  |
|  | M74 | Special technical services | 7 |  |
|  | N77 | Ecological protection and environmental management industry | 24 |  |
|  | N78 | Public facilities management | 7 |  |
|  | Q83 | Health | 30 |  |
|  | R85 | Journalism and publishing | 33 |  |
|  | R86 | Production of radio, TV, film, and recording | 17 |  |
|  | S90 | Comprehensive | 37 |  |
| **Total** |  |  |  | **4844** |
