# Supplementary material for: Diversity of returnee executives’ foreign experience and corporate social responsibility performance
Source: PLoS One. 2024 Apr 1;19(4):e0300262. doi: 10.1371/journal.pone.0300262 (PMC10984457; doi:10.1371/journal.pone.0300262)
Supplement: Attachment — Submitted filename: Responses to reviewers.docx [file pone.0300262.s005.docx]

Responses to Reviewers’ Comments

**SUGGESTIONS FROM ACADEMIC EDITOR:**

***Thank you for submitting your manuscript to our journal for consideration. We appreciate the time and effort you have put into this research. We have completed the initial review of your manuscript. The topic is relevant and has the potential to contribute valuable insights to the field. However, based on the critiques received from our reviewers, we kindly request that you revise your manuscript to address them. Once these revisions have been made, please resubmit your manuscript for further consideration. We believe that addressing these concerns will help to strengthen your paper and its potential impact on the field. We look forward to receiving your revised manuscript. If you have any questions or concerns, please do not hesitate to contact us.***

**REVIEWER’S RESPONSES TO QUESTIONS:**

***1. Is the manuscript technically sound, and do the data support the conclusions?
The manuscript must describe a technically sound piece of scientific research with data that supports the conclusions. Experiments must have been conducted rigorously, with appropriate controls, replication, and sample sizes. The conclusions must be drawn appropriately based on the data presented.***

***Reviewer #1: Yes***

***Reviewer #2: Yes***

***2. Has the statistical analysis been performed appropriately and rigorously?***

***Reviewer #1: Yes***

***Reviewer #2: No***

**Response:**

Thanks to the reviewer’s reminder. In the revised version, we have implemented the reviewer’s recommendations to critically review and refine our statistical tests.

***3. Have the authors made all data underlying the findings in their manuscript fully available?
The*** [***PLOS Data policy***](http://www.plosone.org/static/policies.action#sharing) ***requires authors to make all data underlying the findings described in their manuscript fully available without restriction, with rare exception (please refer to the Data Availability Statement in the manuscript PDF file). The data should be provided as part of the manuscript or its supporting information, or deposited to a public repository. For example, in addition to summary statistics, the data points behind means, medians and variance measures should be available. If there are restrictions on publicly sharing data—e.g. participant privacy or use of data from a third party—those must be specified.***

***Reviewer #1: Yes***

***Reviewer #2: No***

**Response:**

Thanks to the reviewer’s reminder. Research data is reported in supporting information (S4_ZIP. Research data).

***4. Is the manuscript presented in an intelligible fashion and written in standard English?
PLOS ONE does not copyedit accepted manuscripts, so the language in submitted articles must be clear, correct, and unambiguous. Any typographical or grammatical errors should be corrected at revision, so please note any specific errors here.***

***Reviewer #1: Yes***

***Reviewer #2: Yes***

**COMMENTS TO THE AUTHOR:**

**Reviewer #1:**

***I am delighted to have read the article titled "How to achieve green development? A study on spatiotemporal differentiation and influence factors of green development efficiency in China." This study is highly valuable; however, there are several areas that require attention.***

***1. Firstly, the abstract should clearly describe the purpose or need of the study. It is essential to provide a concise overview of research objectives to engage readers effectively.***

**Response:**

We are grateful to the reviewer for raising this valuable point. We have implemented the recommendation suggested by the reviewer and have rewritten the abstract of this study. The revised abstract is set out below:

Top managers’ past experiences (e.g., foreign experience) significantly impact their decision-making behavior, which may influence firms’ sustainable development. The available literature, focusing on the role of the increase in the number of top executives with foreign experience in corporate social responsibility (CSR), yields mixed results. In order to clarify the ambiguous relationship between executive foreign experience and CSR, we empirically examine the effect of the geographic diversity of top executives’ foreign experience on CSR. Based on a hand-collected dataset of the top management team’s (TMT’s) foreign experience, we demonstrate the positive impact of the geographic diversity of returnee executives’ foreign experience on firms’ CSR using Chinese A-share listed firms from 2009 to 2018. Moreover, this impact is stronger in firms with political connections with the central government and in regions with good market development. Furthermore, the mechanism analysis shows that returnee executives drive firms’ CSR by promoting corporate donations and green innovation. This paper offers clear policy implications by suggesting that hiring returnees with a broad geographic scope of foreign experience as corporate executives is an efficient way to enhance firms’ CSR.

***2. Furthermore, the introduction section appears excessively lengthy. While it is crucial to establish the context and objectives of the study, it would be beneficial to streamline this section. Analyzing previous studies can help identify the research gap, which should be narrowed down to emphasize the novelty and contribution of the current research. Additionally, the research method should be more comprehensively explained in the initial section, providing readers with a clear understanding of the study's approach.***

**Response:**

We are grateful to the reviewer for raising this valuable point. We have now implemented the reviewer’s suggestion. We have streamlined the “Introduction” section in several ways in the revised version.

First, we reviewed the existing literature on returnee executives and corporate social action to identify the research gap in this paper. Overall, the research on returnee executives and corporate social performance is mixed. On the one hand, research suggests that returnee executives benefit firms’ CSR initiatives [1]. On the other hand, it has been suggested that these executives’ foreign experiences may hinder the company’s efforts in social action [2]. These mixed findings produced a gap for our study. Moreover, the existing literature focuses on the number of returnee executives while ignoring the characteristics (e.g., geographic diversity ) of executive foreign experience. Prior studies show that geographic diversity influences returnee executives’ decision-making behavior, thus leading to distinct organizational outcomes [3-5]. Thus, exploring the impact of the geographic diversity of returnee executives’ foreign experience on CSR may help to clarify these mixed findings.

Second, we further articulated the contribution of this study to the existing literature. First of all, this study helps clarify the above-mixed findings by demonstrating the positive impact of returnee executives’ geographic diversity on firms’ CSR performance. Besides, this paper enhances comprehension of international knowledge transfer’s organizational (i.e., political connections) and institutional (i.e., regional market development) boundary conditions. In addition, unlike previous studies concentrating on the direct impact of returnees on corporate sustainable development, this research reveals that returnee talents indirectly affect firms’ social performance by promoting corporate donations and green innovations.

Third, we added the research methods used in the empirical analysis of this study. Specifically, we used ordinary least squares (OLS) regressions to test the main hypotheses. Then, we employed a heteroskedasticity-based identiﬁcation approach [6] to mitigate endogenous concerns. Finally, we conducted several robustness checks to validate our findings. Overall, the results of these checks support our conclusions.

Please see the “Introduction” section in the revised manuscript for details (pages 2-4, lines 37-81, in the revised version).

***3. Regarding the results, although they are relevant and adequately discussed, they lack strong support from significant and recent literature. It would be advantageous to include more recent research articles to enhance the findings' credibility. Incorporating up-to-date sources will contribute to the study's relevance and ensure that readers are informed about the latest developments in the field. Additionally, citing other pertinent and recent sources can strengthen the study's arguments and conclusions. I suggest considering another relevant articles (***[***https://doi.org/10.1177/21582440221116113***](https://doi.org/10.1177/21582440221116113)***)*** [***https://doi.org/******10.1016/j.net.2022.05.022.***](https://doi.org/10.1016/j.net.2022.05.022.)  ***By addressing these concerns and incorporating the suggested improvements, the article will become more concise, up-to-date, and impactful.***

**Response:**

We are grateful to the reviewer for raising this valuable point. In the revised version, we have added recent literature to support our results. Please see the “Results” section in the revised manuscript for details (pages 18, lines 303-323, in the revised version). The papers [7, 8] recommended by the reviewer gave us ideas for future research. We have cited them in the “Limitations and future research” section (page 38, lines 555-565, in the revised version).

**Reviewer #2:**

**SUMMARY**

***The article is definitely relevant to the readership of PLOS ONE. Thank you for the interesting manuscript. However, I still have some serious concerns before publication. Enclosed I describe the main points. I look forward to receiving the revised manuscript again.***

***1. There are several articles that already address the relationship between foreign experience and CSR, especially in the Chinese context (e.g.;*** [***https://doi.org/10.1007/s10551-016-3416-z,***](https://doi.org/10.1007/s10551-016-3416-z,) [***https://doi.org/10.3389/fenvs.2023.1103394,***](https://doi.org/10.3389/fenvs.2023.1103394,) [***https://doi.org/10.1111/acfi.13137,***](https://doi.org/10.1111/acfi.13137,) [***https://doi.org/10.1186/s11782-017-0012-8,***](https://doi.org/10.1186/s11782-017-0012-8,) [***https://doi.org/10.1007/s10551-021-04977-z,***](https://doi.org/10.1007/s10551-021-04977-z,) [***https://doi.org/10.3390/su132011473,***](https://doi.org/10.3390/su132011473,) [***https://doi.org/10.1111/ajfs.12369***](https://doi.org/10.1111/ajfs.12369)***). Please conduct an extensive literature review, cite all relevant articles (not only the ones suggested above) and clearly elaborate to what extent the submitted article goes beyond the existing state of research. In addition, also review to what extent the findings of this article are in line or divergent with the existing articles.***

**Response:**

We are grateful to the reviewer for raising this valuable point. We conducted an extensive literature search and reviewed the existing literature on returnee executives and corporate social action. In addition to the literature [1, 9-14] suggested by the reviewer, we carefully reviewed the other relevant literature [15-17]. Overall, studies show that returnee executives’ foreign experience significantly affects CSR’s overall performance [1, 9-11, 13] and its components [2, 12, 14, 17, 18]. However, the results of these studies are mixed. Research suggests that returnee executives benefit firms’ CSR initiatives [1], while it has been suggested that these executives’ foreign experiences may hinder the company’s efforts in social action [2]. Moreover, the existing research focuses on the number of returnee executives while ignoring the characteristics (e.g., geographic diversity ) of executive foreign experience. Furthermore, previous studies focusing on the direct impact of returnees on corporate sustainability have neglected their indirect impact on CSR.

With a comprehensive review of the literature on returnee executives and corporate social performance, our study can make several contributions to the literature. Unlike previous studies that focused on the number or proportion of returnee executives, we examine a more important characteristic of returnee executives’ foreign experience, namely geographic diversity. Our findings help clarify the mixed relationship between executives’ foreign experience and firms’ CSR. Besides, unlike previous studies concentrating on the direct impact of returnees on corporate sustainable development, this research reveals that returnee talents indirectly impact corporate social performance by promoting corporate donations and green innovations. For details, please refer to the “Introduction” section in the revised manuscript (pages 2-4, lines 37-81, in the revised version).

***2. Streamline the introduction by addressing fewer topics of limited relevance, such as other effects from foreign experience, and avoiding duplication, such as in lines 74 and 84.***

**Response:**

Thanks to the reviewer’s reminder. We have now implemented the suggestion by the reviewer. We have streamlined the “Introduction” section in the revised version by reducing less relevant topics and removing potential duplication. For details, please refer to the “Introduction” section in the revised manuscript for details (pages 2-4, lines 37-81, in the revised version).

***3. Explain why CSR is poor since the average is less than 60. Is 60 the maximum value? (line 292)***

**Response:**

We are grateful to the reviewer for raising this valuable point. In the previous manuscript, we stated that “…average value of *CSR_Score* is 38.797, which does not reach 60, indicating that the overall quality of Chinese firms’ CSR is poor” (page 15, line 292, in the original version). This statement may be unclear. In response, we fixed this in the revised manuscript. The revised statement is that “…average value of CSR_Score is 38.797, with a total score of 100 points, which does not reach 60, indicating that the overall quality of Chinese firms’ CSR is poor” (page 15, line 289, in the revised version). In addition, we have added that RKS provides CSR scores with a minimum value of 0 and a maximum value of 100 in the “Measurements” section (page 11, lines 229-230, in the revised version). Therefore, there is no doubt that an average score of less than 60 out of 100 reflects poor CSR performance in China.

***4. The definition of „geographic diversity“ is highly relevant for you article. In article you address foreign experience as number of stations in "developed countries". Please specify the list of "developed countries" e.g., in an appendix. Do additional calculations or clarify based on references why the number of stations is particularly relevant for CSR awareness and not rather the number of years spent abroad. Are there minimum periods that had to be spent in a country for it to count as a foreign station? In addition to "developed countries," "developing countries" are also discussed later (line 345). Which countries are we concerned with here?***

**Response:**

We are grateful to the reviewer for raising this valuable point. We have now implemented the suggestions made by the reviewer. We have provided the list of developed and developing countries/regions in the revised S2 Table. Here, we also report them in the table below for ease of reading for reviewers.

**Table III. Developed and developing countries/regions covered by the study.**

| **Developed countries/regions** | **Developing countries/regions** |
| --- | --- |
| Australia | Bangladesh |
| Austria | Barcelona |
| Belgium | Bavaria |
| Canada | Brazil |
| Finland | Cambodia |
| France | Congo |
| Germany | Dubai |
| Hong Kong, China | India |
| Ireland | Indonesia |
| Italy | Iran |
| Japan | Kazakhstan |
| Luxembourg | Kenya |
| Macao, China | Laos |
| Netherlands | Libya |
| New Zealand | Malaysia |
| Norway | Mexico |
| Poland | Mongolia |
| Singapore | Myanmar |
| South Korea | Nigeria |
| Spain | Pakistan |
| Sweden | Philippines |
| Switzerland | Qatar |
| Taiwan, China | Russia |
| United Kingdom | Senegal |
| United States of America | Thailand |
|  | Tunisia |
|  | Turkey |
|  | Turkmenistan |
|  | Uganda |
|  | Venezuela |
|  | Vietnam |
|  | Zimbabwe |

For the following reasons, we argue that the number of stations, rather than the length of time spent abroad, is more conducive to the transfer of CSR knowledge and beliefs for returnee executives. First, exposure in multiple countries helps enhance executives’ values, such as empathy, respect for others, tolerance for differences, and a sense of social responsibility, motivating them to pursue increased corporate social performance [19, 20]. At the team level, when TMT’s foreign experience covers multiple countries, each member contributes their foreign experience to the team’s CSR decisions. Thus, TMTs with broad foreign experience have a more open mind and international awareness, inspiring them to focus on the interests of a wider range of stakeholders.

Second, knowledge and competencies gained from multiple countries have greater causal ambiguity and can be utilized in multiple contexts rather than a single context. Given the different meanings and styles of CSR actions in different institutional contexts, experiential knowledge gained in one environment may not apply to another. By engaging in and processing foreign experience across countries, executives accumulate diverse practical examples and develop increasingly rich mental schemas about CSR disclosure and practices. These schemas align with their values, beliefs, and strategic behaviors [19]. They feed such schemas into collective TMT cognition [21] and guide the TMT’s information processing, problem-solving, and strategic decisions [22-24]. Thus, they may invoke multiple frameworks or schemas when solving problems, leading to divergent thinking within the TMT and the generation of many strategic alternatives.

In addition, broad experience in different countries increases TMT members’ awareness of cross-country differences [25]. It reduces, to some extent, the occurrence of errors in the process of learning and knowledge transfer. The limited scope of foreign experience may increase the risk of learning errors and overconfidence. However, when the TMT has a large scope of foreign experience, each member contributes his or her foreign experience from different countries to the team’s decisions, allowing diverse perspectives, multiple interpretations, and a broader range of issues to be considered [26, 27]. Prior research on teams has shown that the diversity of perspectives and preferences in a team is positively associated with information sharing, discussion, constructive dissent, and reduced probability of groupthink and overconfidence in team decisions [28-31]. Thus, increased information sharing, constructive dissent, and discussion may allow TMTs with extensive foreign experience to more accurately discern which experiential insights they can and cannot draw on. This also helps avoid incorrect analogizing and erroneous transfer of prior learning [32, 33]. Therefore, we believe that TMTs with diverse foreign experience can make high-quality CSR decisions.

Based on the above analysis, this study focuses primarily on the impact of the breadth of foreign experience (i.e., geographic diversity) rather than its duration on CSR. However, this does not mean the length of stay is unimportant. Too short a stay in a country is not conducive to acquiring experiential knowledge. Therefore, in this paper, we only considered countries/regions where the length of stay is at least one year.

***5. Explain how "political connection" is determined and how it is composed of*** ***CentralPc and LocalPC (line 279). As far as I see there is no aggregated defintionof “political connection”. Please also point this out beforehand in the text.***

**Response:**

We are grateful to the reviewer for raising this valuable point. We have now implemented the suggestions made by the reviewer. In the revised manuscript, we provided a clear definition of what types of firms are politically connected and how political connections consist of *CentralPC* and *LocalPC*. Specifically, we defined a firm as politically connected if at least one of its directors or top managers has served as a government official in central or local government agencies [34, 35]. We distinguish between central and local political connections because of differences in motivations, goals, and priorities between central and local government officials [36, 37]. See pages 34-35, lines 469-492 of the revised version for a discussion of the differences in the motivations and goals of central and local government officials.

Central political connection (*CentralPC*) was measured as the number of TMT and board members who have held a national-level principal or deputy position. Local political connection (*LocalPC*) was measured as the number of TMT and board members who have served as local government officials at the division (chu) level or above. Notably, the hierarchy of local officials in China consists of ministry (bu), department (ju), division (chu), section (ke), staff member (keyuan), and clerk (banshiyuan) in descending order. Lower-level government officials, i.e., those below the level of division (chu), are not included in this study because they are not funded through the central financial system and thus tend not to be counted as political elites in China [38]. See page 12, lines 245-258 of the revised manuscript for the definition and measurement of political connections.

***6. Please check if there are interactions between "political connection" and "marketdev". It could be that "marketdev" partially replaces the relevance of "political connection". Please also check whether "political connection" and "marketdev" are really moderating effects or additional main effects.***

**Response:**

We are grateful to the reviewer for raising this valuable point. We have implemented the reviewer’s suggestions and examined the interaction between political connections (CentralPC and LocalPC) and regional market development (MarketDev). The results are shown in the table below. Column (1) shows that MarketDev, CentralPC, and LocalPC have no significant effect on CSR. Column (2) indicates that the interactive term of MarketDev and CentralPC is insignificant. Similarly, column (3) shows that the interactive term of MarketDev and LocalPC is insignificant. These results suggest that the explanation that market development partially replaces political connections’ relevance is invalid. The moderating role of political connections and market development is still supported.

Table 1. The interactions between political connections and regional market development.

|  | (1) | (2) | (3) | (4) |
| --- | --- | --- | --- | --- |
| Geographic diversity | 1.837*** | 1.831*** | 1.866*** | 1.830*** |
|  | (3.151) | (3.169) | (3.172) | (3.170) |
| MarketDev | 0.253 | 0.291 | 0.270 | 0.294 |
|  | (1.121) | (1.242) | (1.188) | (1.255) |
| CentralPC | 0.361 | 0.295 |  | 0.325 |
|  | (0.618) | (0.517) |  | (0.558) |
| MarketDev*CentralPC |  | 0.444 |  | 0.378 |
|  |  | (1.256) |  | (1.005) |
| LocalPC | -0.066 |  | -0.008 | -0.037 |
|  | (-0.487) |  | (-0.060) | (-0.264) |
| MarketDev*LocalPC |  |  | 0.071 | 0.046 |
|  |  |  | (1.225) | (0.746) |
| ROA | 1.996 | 2.060 | 2.232 | 2.159 |
|  | (0.803) | (0.831) | (0.898) | (0.870) |
| Leverage | -4.595** | -4.535** | -4.559** | -4.521** |
|  | (-2.331) | (-2.307) | (-2.305) | (-2.294) |
| Equity concentration | 0.075*** | 0.075*** | 0.074*** | 0.074*** |
|  | (3.126) | (3.163) | (3.080) | (3.123) |
| Firm age | -0.076 | -0.071 | -0.075 | -0.074 |
|  | (-1.083) | (-1.032) | (-1.067) | (-1.053) |
| Firm size | 3.566*** | 3.530*** | 3.575*** | 3.533*** |
|  | (9.623) | (9.766) | (9.716) | (9.529) |
| Board size | 0.183* | 0.177* | 0.191* | 0.188* |
|  | (1.722) | (1.702) | (1.814) | (1.767) |
| Board independence | -2.295 | -2.485 | -2.368 | -2.375 |
|  | (-0.758) | (-0.832) | (-0.780) | (-0.792) |
| Duality | -0.335 | -0.258 | -0.271 | -0.246 |
|  | (-0.534) | (-0.412) | (-0.431) | (-0.394) |
| Female executive | 2.083*** | 2.063*** | 2.095*** | 2.081*** |
|  | (3.369) | (3.334) | (3.381) | (3.361) |
| Executive age | 0.136 | 0.133 | 0.134 | 0.134 |
|  | (1.430) | (1.393) | (1.403) | (1.414) |
| Foreigner | 2.910** | 2.929** | 2.887** | 2.912** |
|  | (2.487) | (2.480) | (2.461) | (2.477) |
| Returnee director | 0.581 | 0.600 | 0.554 | 0.586 |
|  | (0.977) | (1.011) | (0.931) | (0.986) |
| SOE | 1.199 | 1.183 | 1.125 | 1.181 |
|  | (1.562) | (1.545) | (1.462) | (1.540) |
| Constant | -57.042*** | -54.159*** | -55.205*** | -54.368*** |
|  | (-6.908) | (-6.873) | (-6.790) | (-6.654) |
| Obs. | 4844 | 4844 | 4844 | 4844 |
| Industry FE | yes | yes | yes | yes |
| Year FE | yes | yes | yes | yes |
| Adjusted R-squared | 0.371 | 0.372 | 0.372 | 0.372 |
| F | 16.07*** | 16.36*** | 16.19*** | 14.89*** |

Note. Robust t-statistics (in parentheses) are based on the standard errors clustered by firm to address potential serial correlations in the residuals. *** p<0.01, ** p<0.05, * p<0.1.

***7. In table 4, please also provide the measures R squared corrected and the p value to make the models easier to interpret.***

**Response:**

Thanks to the reviewer’s reminder. We have implemented the reviewer’s recommendation and reported the adjusted R-squared and p-value of the models in the last two rows of Table 4 in the revised version. In addition, we also report the adjusted R-square and p-values in Table 6-10.

***8. It seems that "geographic diversity" can hardly explain CSR, the majority seems to be explained by the control variables. Check the R squared only by "geographic diversity" and interpret the results accordingly.***

**Response:**

We are grateful to the reviewer for raising this valuable point. In this revised manuscript, we have examined the model that includes only the Geographic diversity. The results are presented in Model 1 of Table 4 (page 19, line 325, in the revised version). Model 1 demonstrates a significant positive relationship between the geographic diversity of returnee executives and CSR. The value of the adjusted R-squared is 0.182.

***9. Emphasize that H3 is only supported at 10% level (line 318).***

**Response:**

Thanks to the reviewer’s reminder. We have implemented the reviewer’s recommendation and rewritten this part (page 18, lines 318-323, in the revised version). The statement in the previous manuscript may be a lack of rigor. In response, we have corrected it in the revised manuscript. The revised statement is that “…interaction term in Model 6 is only significantly positive at the 10% level, which produces weak evidence for supporting H3”. Although H3 is only supported at the 10% level, the results of the robustness checks indicate that our findings are robust and credible.

***10. Clarify the difference between "******geographic diversity1" and "geographic diversity" (line 339).***

**Response:**

We are grateful to the reviewer for raising this valuable point. Both Geographic diversity1 and Geographic diversity are used to measure the geographic scope of returnee executives’ foreign experience in developed countries/regions. Geographic diversity1 refers to the number of different foreign stations in which all TMT members are involved (page 22, line 345, in the revised version). Geographic diversity1 considers the TMT as a whole. When there are multiple executives with experience in the same country, Geographic diversity1 is increased by only 1. Therefore, this variable is not related to the number of returnee executives. Geographic diversity is defined as the average number of developed countries where returnee executives studied or worked abroad (page 11, line 239, in the revised version). More specifically, Geographic diversity equals the sum of the number of foreign stations for each returnee executive divided by the number of returning executives.

***11. Foreign experience in developing countries seem at least as well suited as foreign experience in developed countries to explain CSR. Check this observation, which foreign experience is more appropriate in comparison, or does it not matter whether the experience is in developing countries or developed countries (line 381, table 8)***

**Response:**

We are grateful to the reviewer for raising this valuable point. This paper argues that experiences in developed countries or regions are better suited than those in developing countries to explain CSR. Table 8 reported the results of robustness checks controlling for executives’ foreign experience in developing regions. Model 1 shows that returnee executives’ foreign experience in developing countries (DevelopingCou) has no significant impact on CSR.

Moreover, in the revised version, we additionally examined the impact of work and education experiences in developing countries on CSR (pages 28-29, lines 391-407, in the revised version). Models 1 and 2 in Table 10 indicate that foreign work experience from developed countries may be more helpful to returnee executives in improving firms’ CSR than foreign education experience gained from developed countries. Furthermore, we find that neither education nor work experience in developing countries has a significant impact on firms’ CSR. Therefore, the results of Table 8 (page 26, line 381, in the revised version) and Table 10 (page 29, line 408, in the revised version) support our claim that experience in developed countries is more appropriate to explain CSR than experience in developing countries.

***12. Numerous calculations are performed on the robustness of the results (line 338 ff.). The results of all tables (compared to baseline) should each be briefly described.***

**Response:**

Thanks to the reviewer’s reminder. We have implemented the suggestion by the reviewer and briefly stated the results of several robustness checks (pages 22-23, lines 344-371, in the revised version).

***13. What are the definitions of heavily vs. lightly polluting industries. What N is available in the two categories to calculate the models (line 392)?***

**Response:**

We are grateful to the reviewer for raising this valuable point. In the revised manuscript, we have elaborated in more detail on the definitions of heavily and lightly polluting industries (pages 30-31, lines 417-422, in the revised version). We report the number of observations for each industry (N) in S3 Table. Specifically, we define industries in which firms that cause high levels of air and environmental pollution are located as high-polluting industries, and firms that do not cause high levels of air and environmental pollution are located as light-polluting industries. We divided the sample firms according to the Guidelines for Environmental Information Disclosure of Listed Companies issued by China’s Ministry of Environmental Protection in 2010. For heavily polluting industries, 1388 observations were used to calculate the model. For lightly polluting industries, 3456 observations were used for estimation. The sample distribution by heavily and lightly polluting industries is shown in the table below.

**Table V. Sample distribution by heavily and lightly polluting industries.**

| **Industry** | **Industry code** | **Industry name** | **N** | **Total** |
| --- | --- | --- | --- | --- |
| **Heavy-polluted industry** | B06 | Mining and washing of coal | 92 | 1388 |
|  | B07 | Oil and gas extraction | 34 |  |
|  | B08 | Ferrous metal ore mining | 18 |  |
|  | B09 | Nonferrous metal mining | 70 |  |
|  | C15 | Beverage production | 115 |  |
|  | C17 | Textile manufacturing | 53 |  |
|  | C18 | Apparel manufacturing | 42 |  |
|  | C19 | Leather, fur, and feather production | 15 |  |
|  | C22 | Paper production | 44 |  |
|  | C25 | Petroleum processing | 40 |  |
|  | C26 | Chemical materials manufacturing | 250 |  |
|  | C28 | Chemical fiber manufacturing | 35 |  |
|  | C29 | Rubber and plastic production | 53 |  |
|  | C30 | Nonmetallic mineral production | 127 |  |
|  | C31 | Ferrous metal smelting | 131 |  |
|  | C32 | Nonferrous metal smelting | 221 |  |
|  | C33 | Metal product manufacturing | 48 |  |
| **Light-polluted industry** | A01 | Agriculture | 20 | 3456 |
|  | A02 | Forestry | 7 |  |
|  | A03 | Animal husbandry | 20 |  |
|  | A04 | Fishery | 15 |  |
|  | B11 | Mining support industry | 23 |  |
|  | C13 | Agricultural processing | 58 |  |
|  | C14 | Food production | 43 |  |
|  | C21 | Furniture manufacturing | 10 |  |
|  | C23 | Printing and recording | 24 |  |
|  | C24 | Cultural manufacturing | 8 |  |
|  | C27 | Pharmaceutical manufacturing | 278 |  |
|  | C34 | General equipment manufacturing | 141 |  |
|  | C35 | Special equipment manufacturing | 198 |  |
|  | C36 | Automotive manufacturing | 143 |  |
|  | C37 | Transportation equipment | 60 |  |
|  | C38 | Electrical machinery and equipment | 207 |  |
|  | C39 | Computers and communications equipment | 348 |  |
|  | C40 | Instrument manufacturing | 28 |  |
|  | C41 | Other manufacturing | 9 |  |
|  | C42 | Waste utilization industry | 8 |  |
|  | D44 | Production and supply of electric power and heat power | 228 |  |
|  | D45 | Production and Distribution of Gas | 29 |  |
|  | D46 | Water manufacturing and supply | 28 |  |
|  | E48 | Civil engineering construction | 131 |  |
|  | E50 | Architectural decoration and other construction industry | 24 |  |
|  | F51 | Wholesale trade | 147 |  |
|  | F52 | Retail trade | 120 |  |
|  | G53 | Railway transportation | 26 |  |
|  | G54 | Land transportation | 120 |  |
|  | G55 | Ship transportation | 101 |  |
|  | G56 | Flight transportation | 39 |  |
|  | G58 | Loading and transportation agency | 7 |  |
|  | G59 | Storage | 10 |  |
|  | G60 | Post services | 2 |  |
|  | H61 | Accommodation | 10 |  |
|  | I63 | Telecommunications radio and television, and satellite transmission services | 39 |  |
|  | I64 | Internet and related services | 51 |  |
|  | I65 | Software and information technology service | 150 |  |
|  | K70 | Real estate | 335 |  |
|  | L72 | Business services | 54 |  |
|  | M73 | Research and experimental development | 2 |  |
|  | M74 | Special technical services | 7 |  |
|  | N77 | Ecological protection and environmental management industry | 24 |  |
|  | N78 | Public facilities management | 7 |  |
|  | Q83 | Health | 30 |  |
|  | R85 | Journalism and publishing | 33 |  |
|  | R86 | Production of radio, TV, film, and recording | 17 |  |
|  | S90 | Comprehensive | 37 |  |
| **Total** |  |  |  | **4844** |

***14. The paper has many limitations, please describe the main limitations in more detail (line 518).***

**Response:**

We are grateful to the reviewer for raising this valuable point. We describe the limitations of this study in more detail in the revised manuscript. Please see the “Limitations and future research” section for details (pages 37-38, lines 549-573, in the revised version).

***15. Please provide the research data.***

**Response:**

Thanks to the reviewer’s reminder. Research data of this study is reported in supporting information (S4_ZIP. Research data).

**References**

1.	Zhang J, Kong D, Wu J. Doing good business by hiring directors with foreign experience. J Bus Ethics. 2018;153(3):859-76. doi: 10.1007/s10551-016-3416-z.

2.	Zhang L, Xu Y, Chen H. Do returnee executives value corporate philanthropy? Evidence from China. J Bus Ethics. 2022;179(2):411-30. doi: 10.1007/s10551-021-04870-9.

3.	Godart FC, Maddux WW, Shipilov AV, Galinsky AD. Fashion with a foreign flair: Professional experiences abroad facilitate the creative innovations of organizations. Acad Manage J. 2015;58:195-220. doi: 10.5465/AMJ.2012.0575.

4.	Piaskowska D, Trojanowski G, Tharyan R, Ray S. Experience teaches slowly: Non-linear effects of top management teams’ international experience on post-acquisition performance. Br J Manage. 2021. doi: 10.1111/1467-8551.12544.

5.	Mohr A, Batsakis G. The contingent effect of TMT international experience on firms’ internationalization speed. Br J Manage. 2019;30(4):869-87. doi: 10.1111/1467-8551.12293.

6.	Lewbel A. Using heteroscedasticity to identify and estimate mismeasured and endogenous regressor models. J Bus Econ Statist. 2012;30(1):67-80. doi: 10.1080/07350015.2012.643126.

7.	Ali S, Jiang J, Hassan ST, Shah AA. Revolution of nuclear energy efficiency, economic complexity, air transportation and industrial improvement on environmental footprint cost: A novel dynamic simulation approach. Nucl Eng Technol. 2022;54(10):3682-94. doi: 10.1016/j.net.2022.05.022.

8.	Ali S, Naseem MA, Jiang J, Rehman RU, Malik F, Ahmad MI. “How” and “When” CEO duality matter? Case of a developing economy. SAGE Open. 2022;12(3):21582440221116113. doi: 10.1177/21582440221116113.

9.	Zhang YY, Dong LP. Foreign experience of CEO and corporate social responsibility: Evidence from China. Front Environ Sci. 2023;11. doi: 10.3389/fenvs.2023.1103394.

10.	Zheng J, Zhang L. Do venture capitalists with foreign experience drive corporate social responsibility better? Evidence from China. Account Financ. 2023;n/a(n/a). doi: 10.1111/acfi.13137.

11.	Wen W, Song J. Can returnee managers promote CSR performance? Evidence from China. Frontiers of Business Research in China. 2017;11(3). doi: 10.1186/s11782-017-0012-8.

12.	Quan X, Ke Y, Qian Y, Zhang Y. CEO foreign experience and green innovation: Evidence from China. J Bus Ethics. 2023;182(2):535-57. doi: 10.1007/s10551-021-04977-z.

13.	Setiawan D, Brahmana RK, Asrihapsari A, Maisaroh S. Does a foreign board improve corporate social responsibility? Sustainability. 2021;13(20):11473.

14.	Ullah F, Jiang P, Ntim CG, Shahab Y, Jiang X. Female directors’ foreign experience and environmental and sustainable performance*. Asia-Pac J Financ Stud. 2022;51(2):169-93. doi: 10.1111/ajfs.12369.

15.	Lu J, Ren L, Qiao J, Lin W, He Y. Female executives and corporate social responsibility performance: A dual perspective of differences in institutional environment and heterogeneity of foreign experience. Transformations in Business & Economics. 2019;18(2):174-96.

16.	Gao D, Zhao Y, Tian Q. Returnee executives, corporate social responsibility, and stock price synchronicity. Front Psychol. 2022;13:950436-. doi: 10.3389/fpsyg.2022.950436.

17.	Wang Y, Qiu Y, Luo Y. CEO foreign experience and corporate sustainable development: Evidence from China. Bus Strateg Environ. 2022;31(5):2036-51. doi: 10.1002/bse.3006.

18.	Hao Y, Fan C, Long Y, Pan J. The role of returnee executives in improving green innovation performance of Chinese manufacturing enterprises: Implications for sustainable development strategy. Bus Strateg Environ. 2019;28(5):804-18. doi: 10.1002/bse.2282.

19.	Hambrick DC, Mason PA. Upper echelons-The organization as a reflection of its top managers. Acad Manage Rev. 1984;9(2):193-206. doi: 10.2307/258434.

20.	Slater DJ, Dixon-Fowler HR. CEO international assignment experience and corporate social performance. J Bus Ethics. 2009;89(3):473-89. doi: 10.1007/s10551-008-0011-y.

21.	Hambrick DC. Upper echelons theory: An update. Acad Manage Rev. 2007;32(2):334-43.

22.	Dane E. Reconsidering the trade-off between expertise and flexibility: A cognitive entrenchment perspective. Acad Manage Rev. 2010;35(4):579-603. doi: 10.5465/AMR.2010.53502832.

23.	Fee A, Gray SJ, Lu S. Developing cognitive complexity from the expatriate experience: Evidence from a longitudinal field study. International Journal of Cross Cultural Management. 2013;13(3):299-318. doi: 10.1177/1470595813484310.

24.	Maitland E, Sammartino A. Managerial cognition and internationalization. J Int Bus Stud. 2015;46(7):733-60. doi: 10.1057/jibs.2015.9.

25.	Tihanyi L, Ellstrand AE, Daily CM, Dalton DR. Composition of the top management team and firm international diversification. J Manag. 2000;26(6):1157-77. doi: 10.1177/014920630002600605.

26.	Nielsen BB, Nielsen S. Top management team nationality diversity and firm performance: A multilevel study. Strateg Manage J. 2013;34(3):373-82. doi: 10.1002/smj.2021.

27.	Sambharya RB. Foreign experience of top management teams and international diversification strategies of US multinational corporations. Strateg Manage J. 1996;17(9):739-46. doi: 10.1002/(sici)1097-0266(199611)17:9<739::Aid-smj846>3.0.Co;2-k.

28.	Meissner P, Schubert M, Wulf T. Determinants of group-level overconfidence in teams: A quasi-experimental investigation of diversity and tenure. Long Range Plann. 2018;51(6):927-36. doi: 10.1016/j.lrp.2017.11.002.

29.	Cox TH, Blake S. Managing cultural diversity: Implications for organizational competitiveness. Academy of Management Perspectives. 1991;5(3):45-56. doi: 10.5465/ame.1991.4274465.

30.	Schulz-Hardt S, Jochims M, Frey D. Productive conflict in group decision making: Genuine and contrived dissent as strategies to counteract biased information seeking. Organ Behav Hum Decis Process. 2002;88(2):563-86. doi: 10.1016/S0749-5978(02)00001-8.

31.	Scholten L, van Knippenberg D, Nijstad BA, De Dreu CKW. Motivated information processing and group decision-making: Effects of process accountability on information processing and decision quality. Journal of Experimental Social Psychology. 2007;43(4):539-52. doi: 10.1016/j.jesp.2006.05.010.

32.	Gary MS, Wood RE, Pillinger T. Enhancing mental models, analogical transfer, and performance in strategic decision making. Strateg Manage J. 2012;33(11):1229-46. doi: 10.1002/smj.1979.

33.	Gavetti G, Levinthal DA, Rivkin JW. Strategy making in novel and complex worlds: The power of analogy. Strateg Manage J. 2005;26(8):691-712. doi: 10.1002/smj.475.

34.	Du J, Bai T, Chen S. Integrating corporate social and corporate political strategies: Performance implications and institutional contingencies in China. J Bus Res. 2019;98:299-316. doi: 10.1016/j.jbusres.2019.02.014.

35.	Wang Z, Reimsbach D, Braam G. Political embeddedness and the diffusion of corporate social responsibility practices in China: A trade-off between financial and CSR performance? J Clean Prod. 2018;198:1185-97. doi: 10.1016/j.jclepro.2018.07.116.

36.	Bai C-E, Lu J, Tao Z. The multitask theory of state enterprise reform: Empirical evidence from China. Am Econ Rev. 2006;96(2):353-7. doi: 10.1257/000282806777212125.

37.	Cai H, Treisman D. Did government decentralization cause China’s economic miracle? World Polit. 2006;58(4):505-35.

38.	Haveman HA, Jia N, Shi J, Wang Y. The dynamics of political embeddedness in China. Adm Sci Q. 2017;62(1):67-104. doi: 10.1177/0001839216657311.
