# Supplementary material for: Diversity of returnee executives’ foreign experience and corporate social responsibility performance
Source: PLoS One. 2024 Apr 1;19(4):e0300262. doi: 10.1371/journal.pone.0300262 (PMC10984457; doi:10.1371/journal.pone.0300262)
Supplement: Attachment — Submitted filename: Responses to reviewers.docx [file pone.0300262.s006.docx]

Responses to Reviewers’ Comments

**REVIEWER’S RESPONSES TO QUESTIONS:**

***1. If the authors have adequately addressed your comments raised in a previous round of review and you feel that this manuscript is now acceptable for publication, you may indicate that here to bypass the “Comments to the Author” section, enter your conflict of interest statement in the “Confidential to Editor” section, and submit your "Accept" recommendation.***

***Reviewer #1: All comments have been addressed***

***Reviewer #2: (No Response)***

***2. Is the manuscript technically sound, and do the data support the conclusions?***

***The manuscript must describe a technically sound piece of scientific research with data that supports the conclusions. Experiments must have been conducted rigorously, with appropriate controls, replication, and sample sizes. The conclusions must be drawn appropriately based on the data presented.***

***Reviewer #1: Yes***

***Reviewer #2: Yes***

***3. Has the statistical analysis been performed appropriately and rigorously?***

***Reviewer #1: Yes***

***Reviewer #2: No***

**Response:**

Thanks to the reviewer’s reminder. We have implemented the recommendation suggested by the reviewer to ensure the appropriateness and rigour of statistical analysis.

***4. Have the authors made all data underlying the findings in their manuscript fully available?***

***The PLOS Data policy requires authors to make all data underlying the findings described in their manuscript fully available without restriction, with rare exception (please refer to the Data Availability Statement in the manuscript PDF file). The data should be provided as part of the manuscript or its supporting information, or deposited to a public repository. For example, in addition to summary statistics, the data points behind means, medians and variance measures should be available. If there are restrictions on publicly sharing data—e.g. participant privacy or use of data from a third party—those must be specified.***

***Reviewer #1: Yes***

***Reviewer #2: Yes***

***5. Is the manuscript presented in an intelligible fashion and written in standard English?***

***PLOS ONE does not copyedit accepted manuscripts, so the language in submitted articles must be clear, correct, and unambiguous. Any typographical or grammatical errors should be corrected at revision, so please note any specific errors here.***

***Reviewer #1: Yes***

***Reviewer #2: Yes***

**COMMENTS TO THE AUTHOR:**

**Reviewer #1:**

***In light of the fact that the authors have completed all the revisions and addressed all the questions raised during the review process, the paper is ready for publication. The authors have provided a comprehensive response to the reviewer's comments, and it is clear that they have taken the reviewer's suggestions into consideration. Furthermore, the paper has been thoroughly edited and proofread, and all of the technical issues have been addressed.***

**Reviewer #2:**

***Many thanks for the extensive revisions, the level of the article has definitely been raised. I support the publication of the article in the future!***

***However, I have one last comment on Table S2 and the classifications made. The regions are largely at country level and that is exactly how it should be. In some cases, however, there is a deviation from this, which should be avoided as it leads to errors. For example, Barcelona and Bavaria are shown as developing countries/regions. However, Spain and Germany are shown in the list of developed countries/regions. Corrections should be made here and then the calculation should be renewed once with the changed data, even if it should not change the results significantly.***

**Response:**

Thanks to the reviewer’s reminder. We apologize for our carelessness. We have implemented the recommendation suggested by the reviewer. First, we have fixed a bug in the S2 Table regarding the categorization of regions. We reclassified Barcelona as Spain and Bavaria as Germany. In addition, Dubai has been categorized as the United Arab Emirates. These changes have been made to ensure that, as far as possible, the regions are at the country level. The corrected list of developed and developing countries/regions is set out below:

**Table III. Developed and developing countries/regions covered by the study.**

| **Developed countries/regions** | **Developing countries/regions** |
| --- | --- |
| Australia | Bangladesh |
| Austria | Brazil |
| Belgium | Cambodia |
| Canada | Congo |
| Finland | India |
| France | Indonesia |
| Germany | Iran |
| Hong Kong, China | Kazakhstan |
| Ireland | Kenya |
| Italy | Laos |
| Japan | Libya |
| Luxembourg | Malaysia |
| Macao, China | Mexico |
| Netherlands | Mongolia |
| New Zealand | Myanmar |
| Norway | Nigeria |
| Poland | Pakistan |
| Singapore | Philippines |
| South Korea | Qatar |
| Spain | Russia |
| Sweden | Senegal |
| Switzerland | Thailand |
| Taiwan, China | Tunisia |
| United Kingdom | Türkiye |
| United States of America | Turkmenistan |
|  | Uganda |
|  | United Arab Emirates |
|  | Venezuela |
|  | Vietnam |
|  | Zimbabwe |

Second, we have also updated the executive-level data. S2 Table indicates that summary statistics of the executive-level data (Table II in S2 Table) have little changes.

Finally, we conducted the statistical analysis using the changed data. We reexamined all the estimates in this paper, including descriptive statistics, correlation, and regression analysis. Although the results in the revised version have changed slightly compared with the previous version, this has not significantly influenced the results. Our main findings are still supported. The changed data was included in S4_ZIP of the revised version.
